# S-Nitrosylation of G protein-coupled receptor kinase 6 and Casein kinase 2 alpha modulates their kinase activity toward alpha-synuclein phosphorylation in an animal model of Parkinson’s disease

**DOI:** 10.1371/journal.pone.0232019

**Published:** 2020-04-28

**Authors:** Weiwei Wu, Chun Chau Sung, Peichun Yu, Jiahua Li, Kenny K. K. Chung

**Affiliations:** Division of Life Science, State Key Laboratory of Molecular Neuroscience, The Hong Kong University of Science and Technology, Hong Kong, China; Louisiana State University Health Sciences Center, UNITED STATES

## Abstract

Parkinson’s disease (PD) is a common neurodegenerative disorder which is mostly sporadic but familial-linked PD (FPD) cases have also been found. The first reported gene mutation that linked to PD is α-synuclein (α-syn). Studies have shown that mutations, increased expression or abnormal processing of α-syn can contribute to PD, but it is believed that multiple mechanisms are involved. One of the contributing factors is post-translational modification (PTM), such as phosphorylation of α-syn at serine 129 by G-protein-coupled receptor kinases (GRKs) and casein kinase 2α (CK2α). Another known important contributing factor to PD pathogenesis is oxidative and nitrosative stress. In this study, we found that GRK6 and CK2α can be S-nitrosylated by nitric oxide (NO) both in vitro and in vivo. S-nitrosylation of GRK6 and CK2α enhanced their kinase activity towards the phosphorylation of α-syn at S129. In an A53T α-syn transgenic mouse model of PD, we found that increased GRK6 and CK2α S-nitrosylation were observed in an age dependent manner and it was associated with an increased level of pSer129 α-syn. Treatment of A53T α-syn transgenic mice with Nω-Nitro-L-arginine (L-NNA) significantly reduced the S-nitrosylation of GRK6 and CK2α in the brain. Finally, deletion of neuronal nitric oxide synthase (nNOS) in A53T α-syn transgenic mice reduced the levels of pSer129 α-syn and α-syn in an age dependent manner. Our results provide a novel mechanism of how NO through S-nitrosylation of GRK6 and CK2α can enhance the phosphorylation of pSer129 α-syn in an animal model of PD.

## Introduction

Parkinson’s disease (PD) is a common neurodegenerative disorder marked by impaired movement in association with a selective loss of dopaminergic neurons in the midbrain [1–3]. PD is largely sporadic but familial cases are also found [1–3]. For instance, mutation in α-synuclein (α-syn) was first mapped in an Italian family with history of familial form of PD (FPD) [4]. This soon has led to the finding that α-syn is a major component of Lewy bodies (LBs), which is a classical pathological hallmark of PD [5, 6]. The exact mechanism of how α-syn contributes to the formation of LBs and pathogenesis of PD has since become the focus of PD research [7–11]. Studies have suggested that formation of oligomeric species, fibrils and aggregated form of α-syn are the prime suspects in causing the degeneration of dopaminergic neurons in PD [12, 13]. However, the mechanism of the formation of these toxic α-syn species still remains unclear [14, 15]. Studies have shown that post-translational modification (PTM) such as nitration, phosphorylation or dopamine adduct can increase α-syn’s propensity to form toxic oligomeric species [16–18]. One of the most studied α-syn PTM is phosphorylation and the major phosphorylation residue of α-syn is ser129 (S129) [17–21]. Several kinases have been shown to phosphorylate α-syn which includes G-protein-coupled receptor kinases (GRKs), polo-like kinases (PLKs) and casein kinases (CKs) [22–27]. For instance, studies have shown that both GRK6 and CK2 targets α-syn for S129 phosphorylation [21, 27–29] and large proportion of the α-syn in PD brain samples are found to be phosphorylated at S129 whereas in control, most α-syn is not phosphorylated [19, 30]. These results suggested that the level of phosphorylated α-syn is playing an important role in the neurodegeneration in PD. Another major contributor for PD is believed to be nitrosative stress as studies have shown that increased oxidative stress is one of the common pathogenic features observed in PD patients [31, 32]. In particular, previous studies have shown that increased nitrosative stress is prominent in PD pathogenesis and has been shown to impair a number of pathways that protect dopaminergic neurons against toxic insults [33–36]. For example, nitric oxide (NO) mediating modifications of Parkin, XIAP and CDK5 through S-nitrosylation have been shown to affect their role in neuronal survival [33–38]. In this study, we found that GRK6 and CK2α can be S-nitrosylated both *in vitro* and *in vivo*. S-nitrosylation of GRK6 and CK2α enhanced their kinase activity towards the phosphorylation of α-syn at S129. In an A53T α-syn transgenic mouse model of PD, we found that increased GRK6 and CK2α S-nitrosylation was observed in an age dependent manner and it was associated with increased levels of pSer129 α-syn. Treatment of A53T α-syn transgenic mice with Nω-Nitro-L-arginine (L-NNA) significantly reduced the S-nitrosylation of GRK6 and CK2α in the brain. Finally, deletion of neuronal nitric oxide synthase (nNOS) in A53T transgenic mice reduced the levels of pSer129 α-syn and α-syn in an age dependent manner. Our results provide a novel mechanism of how NO through S-nitrosylation of GRK6 and CK2α can enhance the phosphorylation of pSer129 α-syn in a mouse model of PD.

## Materials and methods

### Chemicals, plasmids and antibodies

All chemicals were purchased from (Sigma-Aldrich St. Louis, MO) unless otherwise stated. GRK6 was cloned from Super Script human brain cDNA library (Invitrogen, Carlsbad, CA). Full-length GRK6 was then cloned into FLAG-tagged pCMV-Tag-2B using EcoRI and SalI for cellular expression of GRK6. GRK6 mutants were generated by site-directed mutagenesis using fusion PCR method and cloned into the same vector and verified by DNA sequencing. Plasmids containing the coding sequence of CK2α and CK2β were kind gifts from Prof Robert Qi, The Hong Kong University of Science and Technology. CK2α and CK2β were sub-cloned into myc-tagged and HA-tagged pRK5 vector for cell culture studies and pET28c vector for recombinant protein purification. Site-directed mutagenesis of Cys147 and Cys220 to serine of CK2α was performed by two-steps fusion PCR. WT α-synuclein (α-syn) was cloned into pRK5 and pET28c vectors. Sequence integrity and all mutations were verified by sequencing. Antibodies used for Western blot analysis included the followings: rabbit anti-α-syn (SC-7011-R, Santa Cruz Biotechnology, Dallas, TX), mouse anti-β-actin (A5316, Sigma, St. Louis, MO), mouse anti-myc (11667203001, Roche, Indianapolis, IN), mouse anti-HA (11666606001, Roche, Indianapolis, IN), rabbit anti-phospho-specific ser129 α-syn antibody (ab168381, Abcam, Cambridge, UK), mouse anti-GAPDH (PA1-987, Invitrogen, Thermo Fisher Scientific, Waltham, MA), rabbit anti-CK2 alpha (ab236664, Abcam, Cambridge, UK), mouse anti-GRK6 (05–466, EMD Millipore, Billerica, MA).

### Expression and purification of recombinant proteins

Recombinant his-tagged CK2α and α-syn in pET-28c were transformed into Rosetta (DE3) pLys *Escherichia coli* (Novagen, Billerica, MA). Bacterial culture with expression plasmid was grown until linear phase (0.6 OD) and then induced to express the protein by 0.2 mM IPTG at 18°C overnight for 20 hours. The his-tagged recombinant proteins were purified using Ni-NTA Sepharose (GE Healthcare, Piscataway, NJ) according to manufacturer's instructions.

### Preparation of S-nitrosoglutathione

S-nitrosoglutathione (GSNO) was prepared according to Cook [39]. Briefly, 100 mM glutathione (GSH) were reacted with an equal molar of NaNO_2_ in an acidic environment for 10 minutes at room temperature in darkness. NaOH was then added to neutralize the solution and GSNO was precipitated with pre-chilled acetone at –20°C. After precipitation, GSNO pellet was wash and dissolved in MilliQ H_2_O. The concentration of GSNO was determined by spectroscopy at the wavelength of 334 nm with extinction coefficients of ε_334_ = 900 M^-1^ cm^-1^ as described [39]. For all the experiments using GSNO, GSNO was prepared freshly at the day of experiment.

### Biotin-switch assay

Biotin-switch assay was performed as described previously [40]. Cell transfected with desired expression plasmids were lysed in HENT buffer (250 mM HEPES, 1 mM EDTA, 0.1 mM Neocuproine, 1% Triton X-100, 1 mM Aprotinin, 1 mM Leupeptin, 1 mM Benzamidine, 10 mM PMSF) at 4°C. The cell lysate was treated with GSH or NO donor GSNO (250 μM) at room temperature for 15 min. Samples were then passed through the G25 Sephadex spin column (GE Healthcare, Piscataway, NJ) to remove excess GSNO or GSH. Cell lysate was then incubated with 10 mM methanethiosulfonate (MMTS) (Thermo-Fisher, Waltham, MA) at 50°C for 25 minutes and passed through G25 Sephadex spin column to remove excess MMTS. Samples were then incubated with 10 mM ascorbate and 0.4 mM biotin-HPDP (Thermo-Fisher, Waltham, MA) for 1 hour at room temperature with rotation. Excessive biotin-HPDP was removed by acetone precipitation. The protein pellet was then washed 3 times with 75% acetone and re-suspended in 400 μl of HENs buffer (250 mM HEPES, 1 mM EDTA, 0.1 mM Necuproine, 1% SDS). The biotinylated samples were incubated with 50 μl Neutravidin-agarose (Thermo-Fisher, Waltham, MA) for 1 hour at room temperature. The agarose was then pelleted by centrifugation and washed five times with neutralization buffer (20 mM HEPES pH 8.0, 100 mM NaCl, 1 mM EDTA, 0.5% Triton X-100) with 0.6 M NaCl. Samples were then eluted by reducing SDS-PAGE sample buffer and subjected to Western blot analysis. For *in vivo* brain sample biotin switch assay, brain tissues were weighed and homogenized in lysis buffer (HEN buffer with 1% Triton, 1 mM Aprotinin, 1 mM Leupetin, 1 mM Benzamidine, 10 mM PMSF, 20 mM PNPP, 20 mM β-glycerophosphate, 100 mM sodium fluoride; 1 ml lysis buffer per 100 mg tissue). Samples were then centrifuged at 14,800 g for 15 min to separate supernatant and pellet. Protein concentrations were determined by BCA protein assay kit (Thermo-Fisher, Waltham, MA) and samples were subjected to standard biotin-switch assay as described.

### Cell-based *in vitro* GRK6 and CK2α kinase assay

Human wild type α-syn (pRK5 α-syn) together with different amount of either N-terminal FLAG-tagged GRK6 (pCMV-Tag2B-GRK6) or N-terminal myc-tagged CK2α (pRK5-myc-CK2α) was transfected to HEK293T cells in a 6-well plate. Empty vectors (pCMV-Tag2B or pRK5) were added to equalize the amount of plasmid to a final of 1 μg per well. 48 hours after transfection, the cells were lysed in PBS supplied with 1% Triton X-100, 1 mM EDTA, protease inhibitors (1 mM Leupeptin, 1 mM Aprotinin, 1 mM Benzamidine and 10 mM PMSF) and phosphatase inhibitors (50 mM NaF and 1 mM NaVO_4_). After centrifuged in 4°C for 15 minutes at 14,800 g to pellet down the nuclei and debris, the supernatant was subjected to Western blot analysis. Antibodies used for Western blot analysis included the followings: rabbit anti-α-syn (SC-7011-R, Santa Cruz Biotechnology, Dallas, TX), rabbit anti-phospho-specific ser129 α-syn antibody (ab168381, Abcam, Cambridge, UK), rabbit anti-CK2 alpha (ab236664, Abcam, Cambridge, UK), mouse anti-GRK6 (05–466, EMD Millipore, Billerica, MA), mouse anti-β-actin (A5316, Sigma, St. Louis, MO).

### *In vitro* GRK6 kinase assay

N-terminal FLAG-tagged GRK6 or its mutant was expressed in HEK293T cell. 48 hours after transfection, the cells were lysed in lysis buffer containing 50 mM Tris, 150 mM NaCl, 1 mM EDTA, 1 mM DTT, 1% Triton plus protease inhibitors (1 mM Leupeptin, 1 mM Aprotinin, 1 mM Benzamidine and 10 mM PMSF). After centrifuged in 4°C for 5 minutes at 14,800 g to pellet down the nuclei and debris, the supernatant was subjected to immunoprecipitation using protein-A agarose (GE Healthcare, Piscataway, NJ) conjugated anti-FLAG M2 monoclonal antibody (Sigma-Aldrich) with rotation in the cold room for 2 hours. The beads were then washed two times with lysis buffer plus 0.5 mM NaCl and three times with kinase buffer (20 mM Tris, 10 mM NaCl, 10 mM MgCl_2_ and 1 mM EDTA). Equal amount of beads were aliquoted to 1.5 ml Eppendorf tubes before treated with different concentrations of GSNO for 20 minutes at room temperature with rotation in darkness. Excess GSNO was removed by passing the samples through Sephadex G25 desalting column. The GRK kinase assay was then performed in the kinase buffer containing 100 μM ATP, 1–2 μCi of gamma ^32^P ATP (Perkin Elmer, Waltham, MI) and 1 μg of purified his-tagged human α-syn at 30°C for 20 min with vortex on a Thermomixer R shaker (Eppendorf, NY). The reaction was stopped by adding an equal amount of 2X SDS sample buffer and heated at 95°C for 5 min. The samples were then separated using 12% SDS-PAGE, dried on a Bio-Rad Model 583 gel drier and visualized by autoradiography. The band intensity was quantified with Image J (National Institutes of Health). For the *in vitro* GRK6 kinase activity using pSer129 α-syn phosphor-specific antibody, similar procedures were followed as above except non-radioactive ATP was used in the incubation and pSer129 α-syn phosphor-specific antibody was used to detect phosphorylated α-syn.

### *In vitro* CK2 kinase assay using pSer129 α-syn specific antibody

His-tagged CK2α and its cysteine mutant were purified as described above. Recombinant protein kinases were first incubated with different concentrations of GSNO for 20 minutes at room temperature with rotation in darkness. Excess GSNO was removed by passing the samples through Sephadex G25 desalting column. The kinase assay was then performed in the kinase buffer (20 mM Tris pH 7.5, 10 mM NaCl, 10 mM MgCl_2_ and 1 mM EDTA) containing 100 μM ATP and 1 μg of recombinant his-tagged human α-syn at 30°C for 20 minutes with vortex. The reaction was stopped by adding equal amount of 2X SDS sample buffer. The samples were then subjected to Western blot analysis and phosphorylated α-syn at ser 129 (pSer129 α-syn) was detected by phospho-specific antibody to pSer129 α-syn (Abcam, Cambridge, UK). The band intensity was quantified with Image J software (National Institutes of Health).

### Generation of mice for aging and A53T α-syn transgenic expression study

All animal experiments were conducted according to relevant national and international guidelines. The animal protocols used have been reviewed and approved by the Animal Ethics Committee of The Hong Kong University of Science and Technology University. C57BL/6 wild type (WT), A53T α-syn transgenic line M83 (JAX stock #004479) [41], and nNOS knockout mice (JAX stock #002986) [42] were obtained from the Jackson Laboratory (Bar Harbor, ME). Hemizygous A53T α-syn transgenic mice (A53T -/+) were crossed with hemizygous A53T α-syn transgenic mice to obtain control, hemizygous (A53T -/+) and homozygous (A53T +/+) A53T α-syn transgenic mice. The offspring were then genotyped by quantitative PCR according to the protocol provided by The Jackson Laboratory. Hemizygous A53T α-syn transgenic mice (A53T -/+) were crossed with nNOS heterozygous (nNOS -/+) mice to obtain hemizygous A53T α-syn and double transgenic (hemizygous in A53T α-syn; heterozygous in nNOS (A53T -/+; nNOS -/+)) mice. Only male mice were used in the experiments as from our previous study experience, male mice showed a more consistent phenotype in compared to the female mice [43]. For the aging study, 3 hemizygous A53T α-syn transgenic mice (A53T -/+) in each age groups (6, 9 and 12 months) were randomly selected and sacrificed by cervical dislocation, and the brain tissues were harvested for biochemical analysis. For A53T α-syn transgenic expression study, 3 mice of each genotypes (control, hemizygous (A53T -/+) and homozygous (A53T +/+) A53T α-syn transgenic mice) were generated in the way as in the aging study. At 9 months of age, littermates were randomly selected to receive either Nω-Nitro-L-arginine (L-NNA) (15 mg/kg) or PBS as vehicle control. The dosage used for L-NNA was based on previous studies [44–46]. Both groups of the mice received intraperitoneal injection once a day for a total of four weeks. After four weeks, mice were sacrificed by cervical dislocation and the brain tissues were harvested for biochemical analysis. For the nNOS deletion study, littermates of hemizygous A53T α-syn (A53T -/+), and double transgenic mice (A53T -/+; nNOS -/+) were aged to 6, 9 and 12 months. 3 mice from each genotypes of each age groups were sacrificed by cervical dislocation and the brain tissues were harvested for biochemical analysis.

### Statistical analysis

Data were expressed as SEM, statistical significances were determined by Student’s t-test, one-way or two-way ANOVA with Bonferroni post-hoc test. Data were considered significant different when p-value was less than 0.05.

## Results

### GRK6 S-nitrosylation enhances its kinase activity toward α-syn phosphorylation

**Fig 1:**
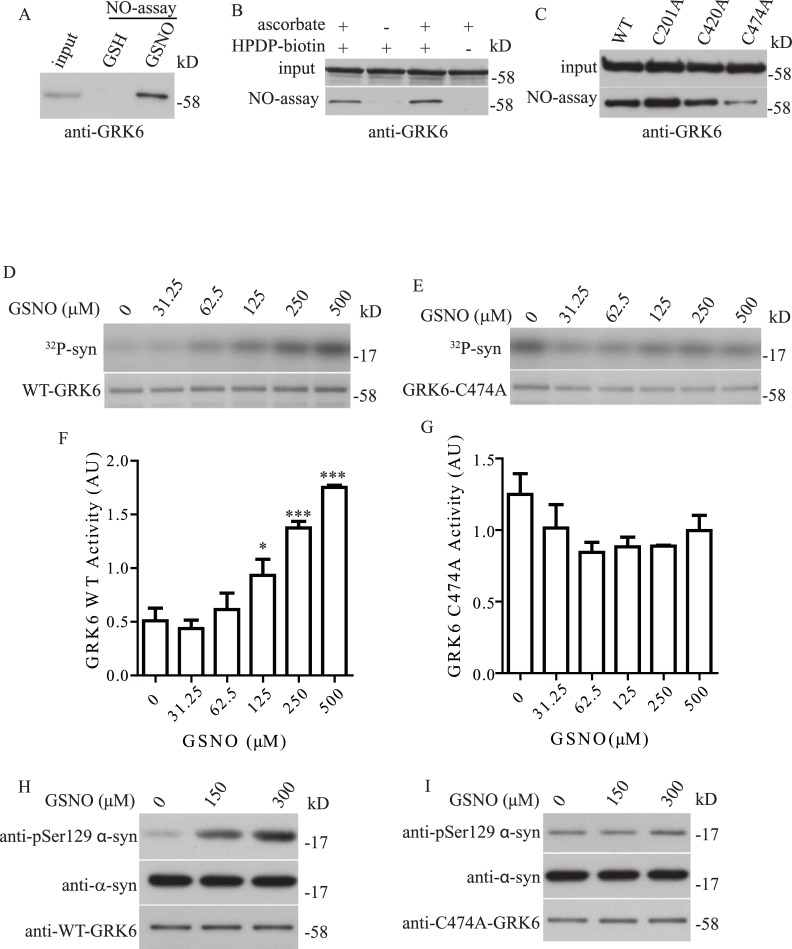
GRK6 S-nitrosylation enhances its kinase activity toward α-syn phosphorylation. **(A)** GRK6 was subject to biotin-switch assay after the treatment of 250 μM of GSH or GSNO. GRK6 was S-nitrosylated after treatment with GSNO. **(B)** Mouse brain lysate was subjected to *in vivo* biotin-switch assay in the presence or absence of ascorbate or HPDP-biotin as controls. GRK6 was S-nitrosylated *in vivo* in the mouse brain. **(C)** GRK6 was S-nitrosylated at C474. WT, C201A, C420A and C474A GRK6 were subjected to biotin-switch assay. Mutation of C474 of GRK6 reduced the level of GRK6 S-nitrosylation. **(D)** GRK6 was subjected to *in vitro* kinase activity assay toward α-syn. After treatment with GSNO, a dose-dependent activation of GRK6 kinase activity toward α-syn was observed. **(E)** GRK6-C474A was subjected to *in vitro* kinase activity assay toward α-syn. After treatment with GSNO, no changes in its kinase activity were observed after treatment with different dosages of GSNO. **(F)** Quantification of GRK6 kinase activity as in (D), (* p < 0.05; *** p < 0.001; # of independent experiments = 3 per each group; one-way ANOVA with Bonferroni post-hoc test). **(G)** Quantification of GRK6-C474A kinase activity as in (E), (# of independent experiments = 3 per each group). **(H)** GRK6 was subjected to *in vitro* kinase assay toward pSer129 α-syn phosphorylation using phospho-specific antibody. Dose-dependent activation of GRK6 kinase activity toward S129 phosphorylation of α-syn was observed after GSNO treatment. **(I)** GRK6-C474A was subjected to *in vitro* kinase activity assay toward pSer129 α-syn phosphorylation after treatment with GSNO. Treatment of GSNO did not affect the kinase activity of GRK6-C474A.

Previous studies have shown that GRK6 can phosphorylate α-syn at S129 [47, 48]. We therefore first tested if GRK6 could enhance the phosphorylation of α-syn at S129 in a dose-dependent manner with a cell-based *in vitro* kinase assay. We transfected HEK293T cells with α-syn and different amounts of GRK6 and performed Western Blot with pSer129 α-syn. Consistent with previous findings, GRK6 enhanced the phosphorylation of α-syn at S129 in a dose-dependent manner (S1 Fig). To determine if GRK6 could be S-nitrosylated *in vitro*, we treated HEK293T cells expressing FLAG-GRK6 with S-nitrosoglutathione (GSNO) or glutathione (GSH) as control. Samples were then subjected to biotin-switch assay and we found that GRK6 was readily S-nitrosylated after treatment with GSNO, but GRK6 S-nitrosylation was not observed in the control (Fig 1A). To determine if GRK6 S-nitrosylation could be detected *in vivo* in mouse brain, we performed *in vivo* biotin-switch assay in the presence or absence of ascorbate and HPDP-biotin in mouse brain lysates. We found that in the absence of ascorbate or HPDP-biotin, GRK6 S-nitrosylation was not observed (Fig 1B), suggesting that GRK6 was S-nitrosylated in vivo in the mouse brain. To determine which cys residue of GRK6 was S-nitrosylated, we generated a number of GRK6 mutants that converted cys residue to ala and determined if these mutations would affect the GRK6 S-nitrosylation. From the screening, we found that mutation of cys 474 of GRK6 reduced its S-nitrosylation (Fig 1C). As in previous studies, S-nitrosylation was shown to able to modulate kinase activity [37, 38], we decided to determine if GRK6 S-nitrosylation could affect its kinase activity towards α-syn. We set up *in vitro* GRK6 kinase activity assay toward α-syn. In this experiment, we first treated GRK6 with different dosages of GSNO and then incubated GRK6 with α-syn for the kinase assay. We found that treatment of GRK6 with GSNO resulted in a dose-dependent increase in GRK6 kinase activity toward the phosphorylation of α-syn (Fig 1D & 1F). This NO induced kinase activity enhancement of GRK6 was depending on cys 474, as mutation of GRK6 cys 474 abrogated the effect of NO treatment in the in vitro kinase assay (Fig 1E & 1G). GRK6 is known to phosphorylate α-syn at S129, to determine if the S-nitrosylation enhanced kinase activity of GRK6 was acting on α-syn S129, we set up GRK6 in vitro kinase activity to assess the GRK6 kinase activity towards α-syn S129 using a well-established pSer129 α-syn phospho-specific antibody [19, 20]. In consistence with the radioactive labelled in vitro GRK6 kinase activity assay (Fig 1D–1G), S-nitrosylation enhanced the GRK6 kinase activity toward the phosphorylation of α-syn (Fig 1H). Mutation of GRK6 at cys 474 abrogated this NO induced enhancement of kinase activity (Fig 1I). These results suggested that S-nitrosylation of GRK6 at cys 474 enhanced its kinase activity toward phosphorylation of α-syn at S129.

### CK2α S-nitrosylation enhances its kinase activity toward α-syn phosphorylation

Previous studies have shown that CK2α can phosphorylate α-syn at S129 [47, 49]. We therefore first tested if CK2α could enhance the phosphorylation of α-syn at S129 in a dose-dependent manner with a cell-based *in vitro* kinase assay. We transfected HEK293T cells with α-syn and different amounts of CK2α and performed Western Blot with pSer129 α-syn. Consistent with previous findings, CK2α enhanced the phosphorylation of α-syn at S129 in a dose-dependent manner (S2 Fig).

**Fig 2:**
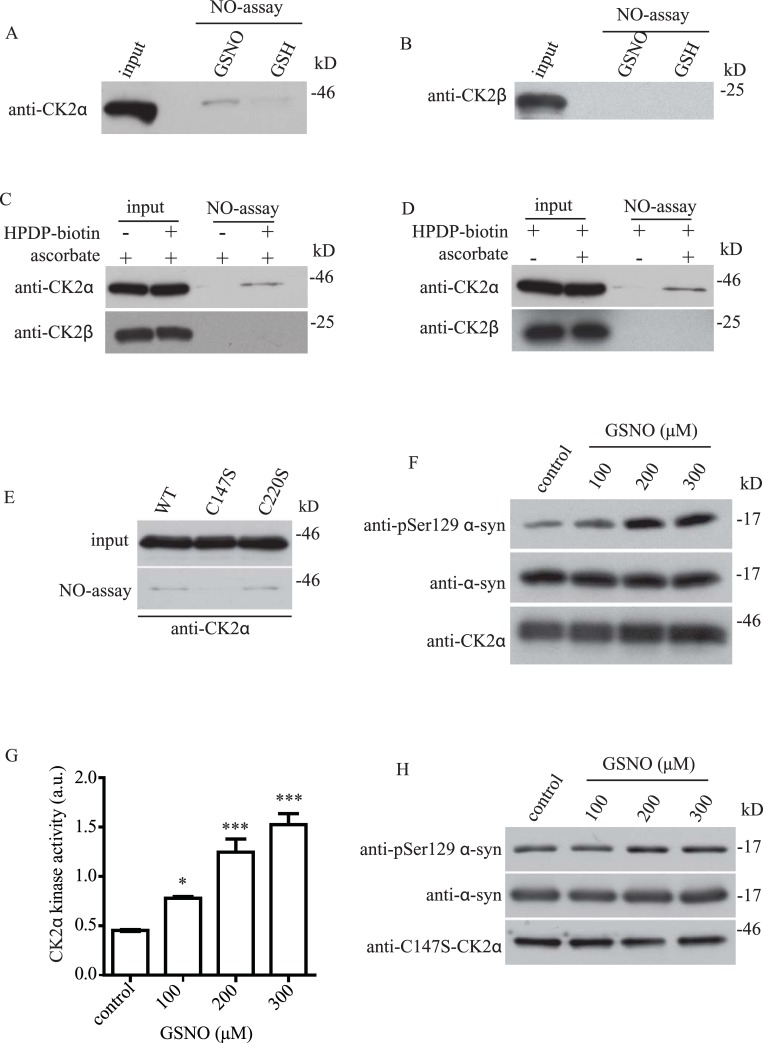
CK2α S-nitrosylation enhances its kinase activity toward α-syn phosphorylation. **(A)** CK2α was subjected to biotin-switch assay after the treatment with 250 μM of GSH or GSNO. CK2α was S-nitrosylated after treatment with GSNO. **(B)** CK2β was subjected to biotin-switch assay after the treatment of 250 μM of GSH or GSNO. CK2β was not S-nitrosylated after the treatment with GSNO. **(C)** Mouse brain lysate was subjected to *in vivo* biotin-switch assay in the presence or absence of ascorbate as control. CK2α was S-nitrosylated *in vivo* in the mouse brain while CK2β was not. **(D)** Mouse brain lysate was subjected to *in vivo* biotin-switch assay in the presence or absence of HPDP-biotin as control. CK2α was S-nitrosylated *in vivo* in the mouse brain while CK2β was not. **(E)** CK2α was S-nitrosylated at C147. WT, C147S and C220S CK2α were subjected to biotin-switch assay. Mutation of C147 of CK2α completely abrogated CK2α S-nitrosylation. **(F)** CK2α was subjected to *in vitro* kinase activity assay toward Ser129 α-syn phosphorylation after treatment with GSNO. Treatment of GSNO led to a dose-dependent activation of CK2α toward α-syn. **(G)** Quantification of CK2α kinase activity as in (F), (* p < 0.05; *** p < 0.001; # of independent experiments = 3 per each group; one-way ANOVA with Bonferroni post-hoc test). **(H)** C147- CK2α was subjected to in vitro kinase activity assay toward pSer129 α-syn phosphorylation after treatment with GSNO. Treatment of GSNO did not affect the kinase activity of C147S-CK2α.

To determine if CK2α could also be S-nitrosylated *in vitro*, we treated HEK293T cells expressing myc-CK2α with S-nitrosoglutathione (GSNO) or glutathione (GSH) as control. Samples were then subjected to biotin-switch assay and we found that CK2α was readily S-nitrosylated after treatment with GSNO, but CK2α S-nitrosylation was not observed in the control (Fig 2A). CK2 is known to exist as a tetramer which consists of two CK2α and two CK2β, we would like to determine if CK2β could also be S-nitrosylated. We treated HEK293T cells expressing HA-CK2β with GSNO and found that CK2β could not be S-nitrosylated (Fig 2B). To determine if CK2α and CK2β S-nitrosylation could be detected in vivo in the mouse brain, we performed *in vivo* biotin-switch assay in the presence or absence of ascorbate and HPDP-biotin in mouse brain lysate. We found that in the absence of ascorbate (Fig 2C) or HPDP-biotin (Fig 2D), CK2α S-nitrosylation was not observed (Fig 2C & 2D), suggesting that CK2α was S-nitrosylated *in vivo* in the mouse brain while CK2β was not (Fig 2C & 2D). Since the CK2α polypeptide sequence only consists of two cysteines (cys 147 and cys 220), we performed mutagenesis and mutated cys 147 and cys 220 to serine to generate C147S and C220S CK2α respectively. To identify which CK2α cys residue was being S-nitrosylated, we performed biotin-switch assay on WT, C147S, and C220S CK2α. We found that mutation of cys 147 to ser specifically abrogated CK2α S-nitrosylation, but mutation of cys 220 to ser had no effect on CK2α S-nitrosylation (Fig 2E). Taken together, these results show that CK2α can be S-nitrosylated both *in vitro* and *in vivo*. As in GRK6, we were interested in if CK2α S-nitrosylation could affect its kinase activity towards α-syn. CK2α has been shown to phosphorylate α-syn at ser129 (pSer129 α-syn) [21]. To determine if CK2α S-nitrosylation could modulate its kinase activity toward α-syn, we set up an *in vitro* CK2α kinase activity assay toward α-syn. In this experiment, we first treated CK2α with different dosages of GSNO and then incubated CK2α with α-syn for the *in vitro* kinase activity assay. We found that treatment of CK2α with GSNO resulted in a dose-dependent increase in CK2α kinase activity toward phosphorylation of α-syn at ser129 (Fig 2F & 2G). This NO-induced kinase activity enhancement of CK2α toward α-syn was dependent on cys 147, as mutation at CK2α cys 147 to ser completely abrogated the effect of NO treatment in the *in vitro* kinase assay (Fig 2H). These results suggested that S-nitrosylation CK2α at cys 147 enhanced its kinase activity toward phosphorylation of α-syn at S129.

### Aging increases GRK6 and CK2α S-nitrosylation in an A53T α-syn transgenic mouse model of PD

**Fig 3:**
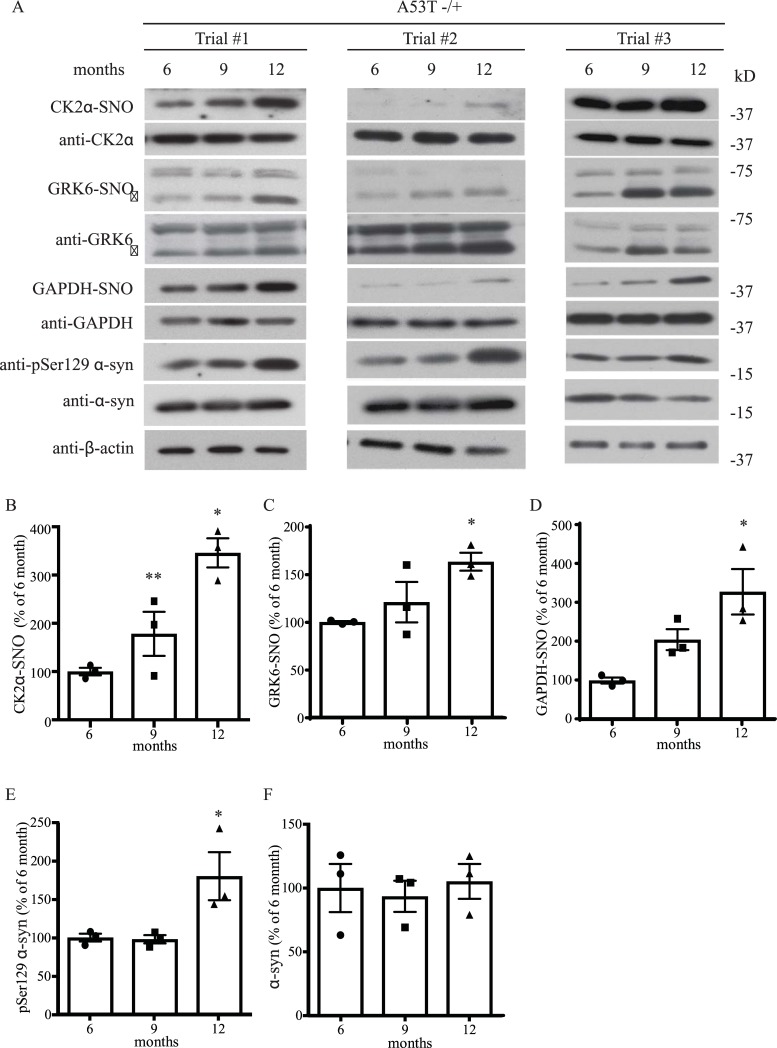
Aging increases GRK6 and CK2α S-nitrosylation in an A53T α-syn transgenic mouse model of PD. **(A)** Hemizygous A53T α-syn transgenic mouse brain samples of 6, 9 and 12 months old were analyzed with *in vivo* biotin-switch assay for GRK6, CK2α and GAPDH. The samples were also subject to Western blot analysis of GRK6, CK2α, GAPDH, pSer129 α-syn and α-syn. (→: GRK6 band) **(B)** Quantification of hemizygous A53T α-syn transgenic mouse brain samples for *in vivo* CK2α S-nitrosylation as in (A) (* p < 0.05, ** p < 0.01; # of animals = 3 for each time point; one-way ANOVA with Bonferroni post-hoc test). (C) Quantification of hemizygous A53T α-syn transgenic mouse brain samples for *in vivo* GRK6 S-nitrosylation as in (A) (* p < 0.05; # of animals = 3 for each time point; one-way ANOVA with Bonferroni post-hoc test). **(D)** Quantification of hemizygous A53T α-syn transgenic mouse brain samples for *in vivo* GAPDH S-nitrosylation as in (A) (* p < 0.05; # of animals = 3 for each time point; one-way ANOVA with Bonferroni post-hoc test). **(E)** Quantification of hemizygous A53T α-syn transgenic mouse brain samples for protein levels of pSer129 α-syn as in (A) (# of animals = 3 for each time point). **(F)** Quantification of hemizygous A53T α-syn transgenic mouse brain samples for protein levels of α-syn as in (A) (# of animals = 3 for each time point).

Since we found that both GRK6 and CK2α could be S-nitrosylated *in vitro* and *in vivo*, we decided to determine if increased S-nitrosylation of them could be observed in a well-characterized A53T α-syn transgenic mouse model of PD [41]. As age is the most significant contributing factor in PD, we design an age-dependent study to determine how GRK6 and CK2α S-nitrosylation would be changed in the A53T α-syn transgenic mouse model of PD. We first generated hemizygous A53T α-syn transgenic mice and these mice were then sacrificed at the age of 6, 9 and 12 months. In this approach, we would be able to determine if aging would be related to changes in GRK6 and CK2α S-nitrosylation. We sacrificed and harvested the brain tissues from these mice at the selected time points and performed *in vivo* biotin-switch assay to analyze GRK6 and CK2α S-nitrosylation in these samples. To include a well-characterized S-nitrosylated protein as a reference, we also analyzed GAPDH S-nitrosylation in these samples [50]. From the Western blot analysis, we did not observe changes in the protein level of GRK6, CK2α and GAPDH in A53T α-syn transgenic mice at different ages (Fig 3A). However, from the *in vivo* biotin-switch assay, we observed an age-dependent increase in the GRK6, CK2α and GAPDH S-nitrosylation in the A53T α-syn transgenic mice (Fig 3A–3D). These results suggested that nitrosative stress could increase during the process of aging and contribute to the pathogenesis of PD. Interestingly we also observed an age-dependent increased in pSer129 α-syn (Fig 3A & 3E), but no change in total α-syn in these samples were observed (Fig 3A & 3F).

### A53T α-syn transgenic expression in mice increases S-nitrosylation of GRK6 and CK2α in the mouse brain and it is dependent on the activity of nitric oxide synthase (NOS)

**Fig 4:**
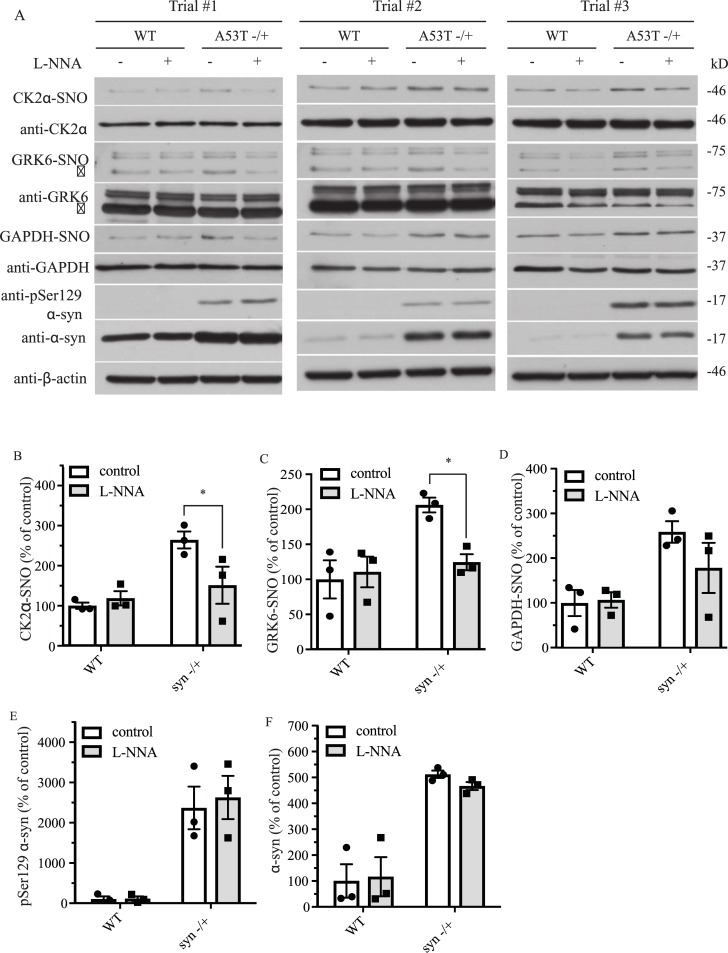
A53T α-syn transgenic expression increases S-nitrosylation of GRK6, CK2α and GAPDH in the mouse brain. **(A)** WT, and hemizygous A53T α-syn transgenic mouse brain samples of 9 months old treated with or without L-NNA were analyzed with *in vivo* biotin-switch assay for CK2α and GAPDH. The samples were also subject to Western blot analysis of CK2α, GAPDH, pSer129 α-syn and α-syn. (→: GRK6 band) **(B)** Quantification of WT and hemizygous A53T α-syn transgenic mouse brain samples for *in vivo* CK2α S-nitrosylation as in (A) (* p < 0.05; no. of animals = 3 in each group; two-way ANOVA with Bonferroni post-hoc test). **(C)** Quantification of WT and hemizygous A53T α-syn transgenic mouse brain samples for *in vivo* GRK6 S-nitrosylation as in (A) (* p < 0.05; # of animals = 3 in each group; two-way ANOVA with Bonferroni post-hoc test). **(D)** Quantification of WT and hemizygous A53T α-syn transgenic mouse brain samples for *in vivo* GAPDH S-nitrosylation as in (A). **(E)** Quantification of WT and hemizygous A53T α-syn transgenic mouse brain samples for protein levels of pSer129 α-syn as in (A) (# of animals = 3 in each group). **(F)** Quantification of WT and hemizygous A53T α-syn transgenic mouse brain samples for protein levels of α-syn as in (A) (# of animals = 3 in each group).

Apart from aging, expression of mutant α-syn like A53T α-syn is known to contribute to PD. We decided to determine if overexpression of A53T α-syn in the mouse transgenic model of PD would enhance GRK6 and CK2α S-nitrosylation. To study the effect of A53T α-syn expression on GRK6 and CK2α, we first generated wild type (WT) and hemizygous A53T α-syn transgenic mice and aged them to 9 months before we performed the experiment. We divided these mice randomly into two groups, for one group, we treated these mice with control saline and for the other group we treated the mice with the NO synthase (NOS) inhibitor Nω-Nitro-L-arginine (L-NNA) for one month. After one month, we sacrificed these mice and harvested the brain tissues to perform *in vivo* biotin-switch assay to analyze the level of GRK6 and CK2α S-nitrosylation. We also analyzed GAPDH S-nitrosylation in these samples as in our previous experiment. In this way, we would be able to determine if transgenic expression of A53T α-syn in mouse brain would affect the S-nitrosylation of GRK6, CK2α and GAPDH and also if NOS activity was involved. Transgenic expression of A53T α-syn clearly increased the amount of pSer129 and total α-syn (Fig 4A, 4E & 4F). Protein levels of GRK6, CK2α and GAPDH were similar in WT and A53T α-syn transgenic mice (Fig 4A). To confirm if this observation was significant, we quantified the results in the WT and hemizygous transgenic mice groups. Transgenic expression of A53T α-syn significantly increased both total (F = 52.26; p < 0.001, two-way ANOVA) and pSer129 α-syn (F = 39.78; p < 0.001, two-way ANOVA) in the transgenic mice (Fig 4A, 4E & 4F). Transgenic expression of A53T α-syn also significantly increased GRK6 (F = 5.01; p < 0.05, two-way ANOVA), CK2α (F = 13.02; p<0.01, two-way ANOVA) and GAPDH (F = 10.94; p < 0.05) S-nitrosylation (Fig 4A–4D). Treatment of the A53T α-syn transgenic mice with L-NNA significantly reduced the A53T α-syn transgenic induced increased in GRK6 and CK2α S-nitrosylation (Fig 4A–4C). L-NNA treatment also reduced GAPDH S-nitrosylation but it was not significant (Fig 4A & 4D). Taken together these results showed that A53T α-syn transgenic expression could increase S-nitrosylation of GRK6 and CK2α and this increase was dependent on the activity of NOS.

### Deletion of neuronal NOS (nNOS) reduces the accumulation of α-syn and pSer129 α-syn in the A53T α-syn transgenic mouse model of PD in an age-dependent manner

**Fig 5:**
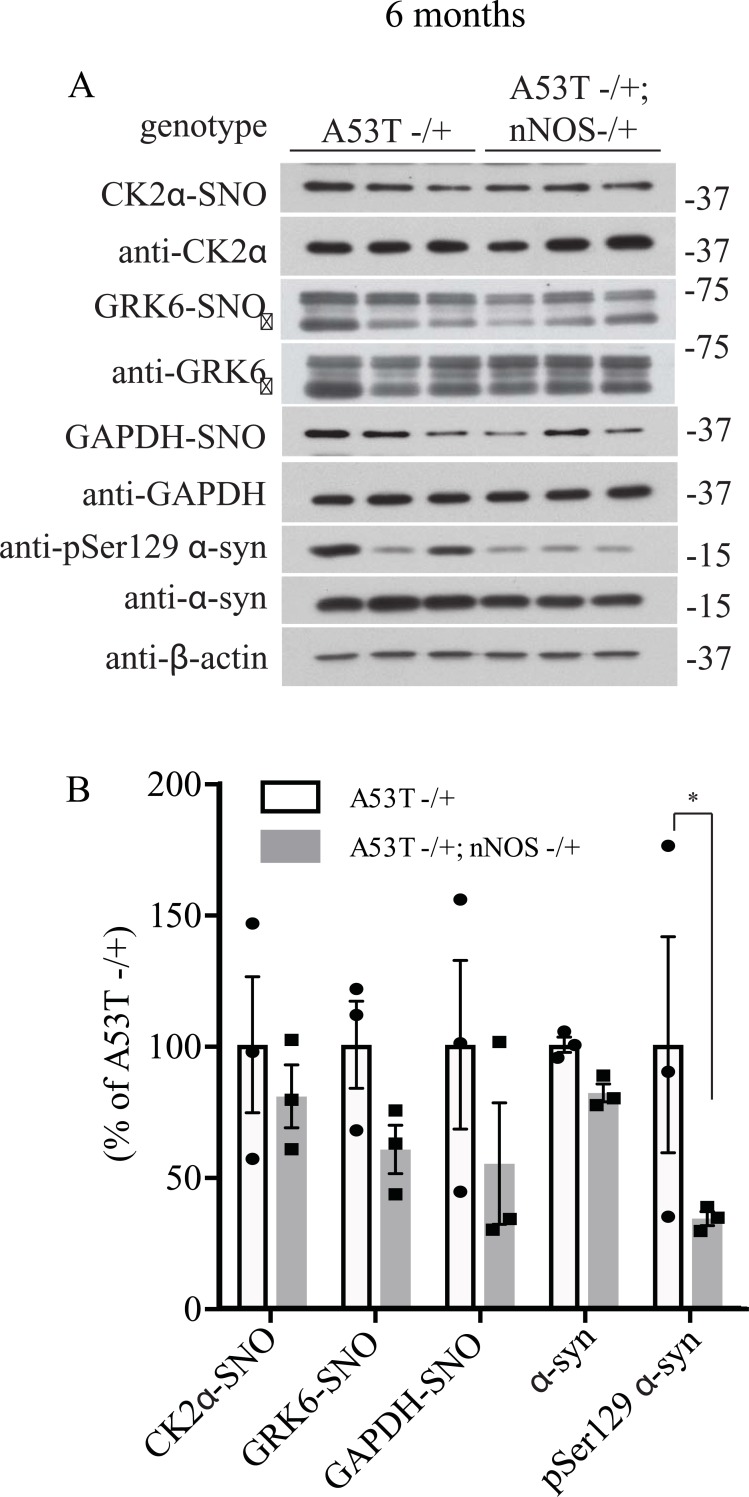
Deletion of neuronal NOS (nNOS) reduces the accumulation of pSer129 α-syn in 6-month-old A53T α-syn transgenic mice. **(A)** Hemizygous A53T α-syn transgenic mouse brain (A53T -/+), and hemizygous A53T α-syn transgenic and nNOS heterozygous knockout (A53T -/+; nNOS -/+) double mutant mouse brain samples at 6-month-old were analyzed with *in vivo* biotin-switch assay for CK2α and GAPDH. The samples were also subject to Western blot analysis of CK2α, GAPDH, pSer129 α-syn and α-syn. (→: GRK6 band) **(B)** Quantification of CK2α, GRK6 and GAPDH S-nitrosylation and protein levels of pSer129 α-syn and α-syn as in (A) (* p<0.05; # of animals = 3 in each group; Student’s t-test).

**Fig 6:**
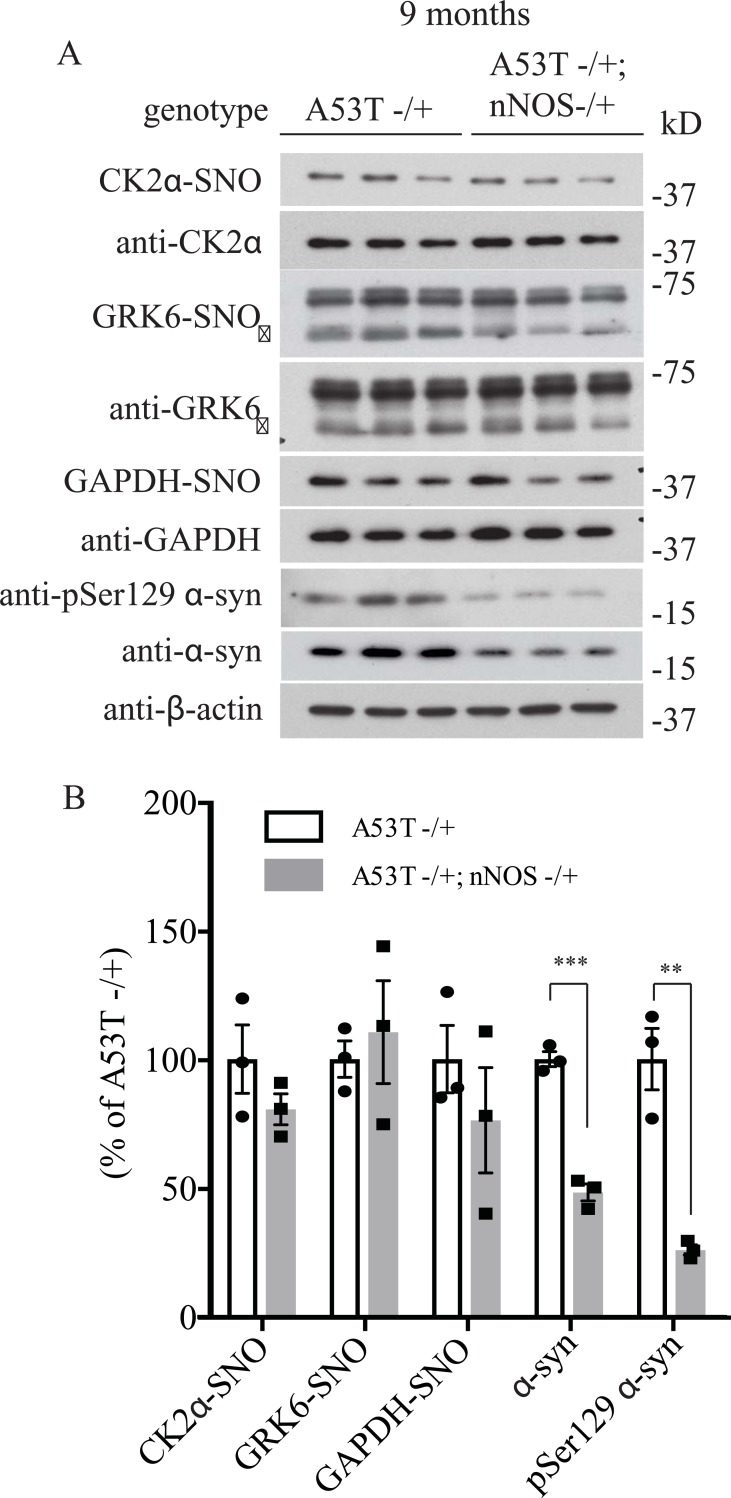
Deletion of neuronal NOS (nNOS) reduces the accumulation of pSer129 α-syn and total α-syn in 9-month-old A53T α-syn transgenic mice. **(A)** Hemizygous A53T α-syn transgenic mouse brain (A53T -/+), and hemizygous A53T α-syn transgenic and nNOS heterozygous knockout (A53T -/+; nNOS -/+) double mutant mouse brain samples at 9-month-old were analyzed with *in vivo* biotin-switch assay for CK2α and GAPDH. The samples were also subject to Western blot analysis of CK2α, GAPDH, pSer129 α-syn and α-syn. (→: GRK6 band) **(B)** Quantification of CK2α, GRK6 and GAPDH S-nitrosylation and protein levels of pSer129 α-syn and α-syn as in (A) (*** p<0.001; ** P<0.01; # of animals = 3 in each group; Student’s t-test).

**Fig 7:**
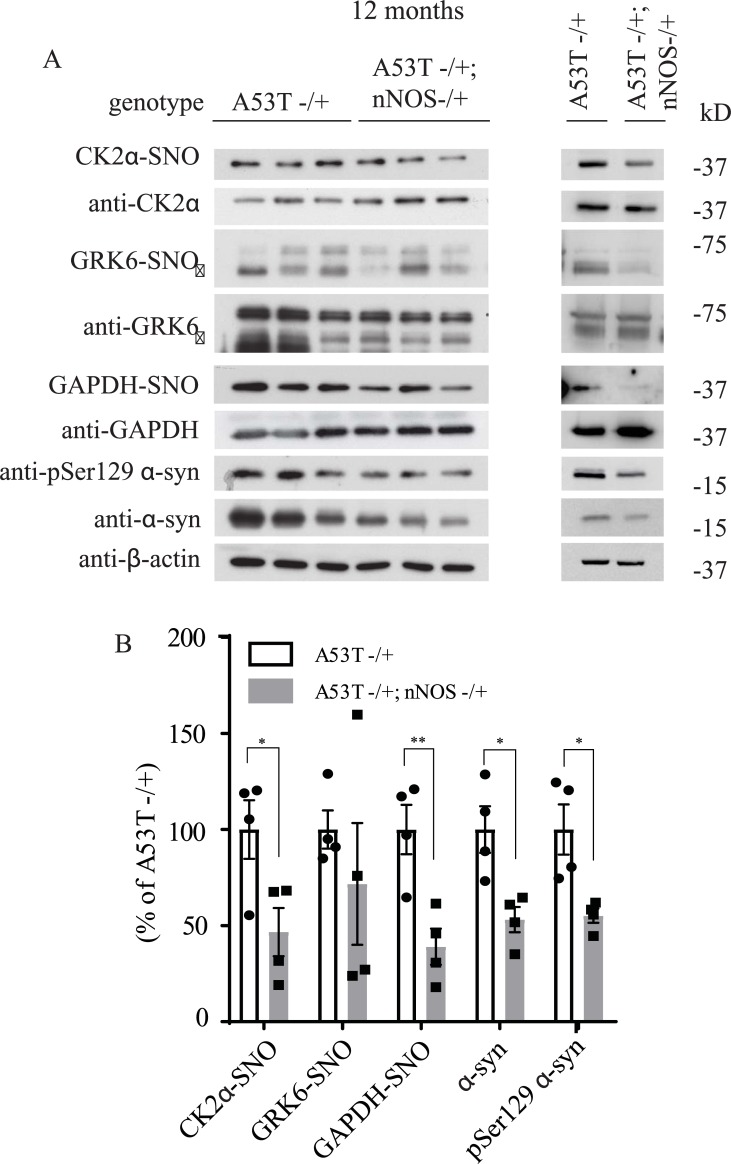
Deletion of neuronal NOS (nNOS) reduces the accumulation of pSer129 α-syn and total α-syn in 12-month-old A53T α-syn transgenic mice. **(A)** Hemizygous A53T α-syn transgenic mouse brain (A53T -/+), and hemizygous A53T α-syn transgenic and nNOS heterozygous knockout (A53T -/+; nNOS -/+) double mutant mouse brain samples at 12-month-old were analyzed with *in vivo* biotin-switch assay for CK2α and GAPDH. The samples were also subject to Western blot analysis of CK2α, GAPDH, pSer129 α-syn and α-syn. (→: GRK6 band) **(B)** Quantification of CK2α, GRK6 and GAPDH S-nitrosylation and protein levels of pSer129 α-syn and α-syn as in (A) (* p<0.05; ** p<0.01; # of animals = 4 in each group; Student’s t-test).

There are three isoforms of NOS in mammals which include inducible NOS (iNOS), endothelial NOS (eNOS) and neuronal NOS (nNOS). nNOS is highly expressed in the brain and therefore we wanted to determine if nNOS was responsible for the increase in S-nitrosylation of GRK6 and CK2α in the A53T α-syn transgenic mice. To test if heterozygous deletion of nNOS would affect the S-nitrosylation of GRK6 and CK2α in the A53T α-syn transgenic mice, we crossed the nNOS knockout mice with the A53T α-syn transgenic to establish mice with genotypes of A53T -/+ and A53T -/+; nNOS-/+. After 6, 9 and 12 months of age, we sacrifice these mice and harvested the brain tissues to perform *in vivo* biotin switch assay to analyze the level of GRK6, CK2α and GAPDH S-nitrosylation. In this way, we would be able to determine if increased S-nitrosylation of GRK6 and CK2α in A53T α-syn transgenic mice was dependent on nNOS. We found that deletion of nNOS did not significantly affect the S-nitrosylation of GRK6, CK2α and GAPDH in 6 and 9 months A53T α-syn transgenic mice (Figs 5A, 5B, 6A & 6B). In contrast, deletion of nNOS was found to significantly decrease the S-nitrosylation of CK2α and GAPDH in the 12 month-old A53T α-syn transgenic mice (Fig 7A & 7B). Interestingly, a significant reduction of pSer129 α-syn was observed in 6-month-old A53T α-syn transgenic mice with the heterozygous deletion of nNOS (A53T -/+; nNOS-/+) (Fig 5A & 5B). In 9-month-old A53T α-syn transgenic mice with the heterozygous deletion of nNOS (A53T -/+; nNOS-/+), both pSer129 α-syn and total α-syn were significantly reduced (Fig 6A & 6B). Similar to the 9-month-old A53T α-syn transgenic mice with the heterozygous deletion of nNOS (A53T -/+; nNOS-/+), both pSer129 α-syn and total α-syn were significantly reduced in the 12-month-old double transgenic mice (Fig 7A & 7B). These results suggest that both aging and NO could modulate the levels of pSer129 α-syn and total α-syn in the mouse brain.

## Discussion

Oxidative stress and α-syn aggregation are known to be the important contributing factors to the pathogenesis of PD. Early studies have shown that nitration of α-syn can enhance α-syn aggregation and formation of LBs [10, 51]. Other studies have also shown that nitrosative stress through S-nitrosylation can contribute to the pathogenesis of PD [32, 52]. For instance, our previous studies have shown that S-nitrosylation of parkin and XIAP can impair their neuroprotection functions and contribute to PD [33–36]. In this study, we found that CK2α and GRK6 could be S-nitrosylated both *in vitro* and *in vivo* and this modification enhanced their kinase activity towards phosphorylation of α-syn at ser129. To determine if S-nitrosylation of CK2α and GRK6 is relevant to PD, we studied the level of S-nitrosylation of CK2α and GRK6 in a well-established A53T α-syn transgenic mouse model of PD [41]. In addition, we also monitored the level of pSer129 α-syn and total α-syn in these mouse brain samples. From our results on aging study, we found that aging increased S-nitrosylation of CK2α, GRK6 and GAPDH in association with the increased in the amount of pSer129 α-syn (Fig 3). This result provides a novel correlation among aging, nitrosative stress and increased accumulation of pSer129 α-syn in the development of PD. To further determine how nitrosative stress can contribute to PD in the A53T α-syn transgenic mouse model, we treated 9 months old mice with or without NOS inhibitor L-NNA for one month. We found that even one month L-NNA treatment could significantly reduce the S-nitroyslation of CK2α and GRK6 (Fig 4). However, we didn’t observe any changes in the level of α-syn and pSer129 α-syn in these mice (Fig 4). It is highly possible that such a short-term inhibition of NOS was not long enough to affect the metabolism of α-syn and pSer129 α-syn through the reduction of CK2α and GRK6 S-nitrosylation. This notion is supported by our aging and double mutant (A53T -/+; nNOS-/+) data in which changes of α-syn and pSer129 α-syn level were not observed until mice were 9 or 12 months old (Figs 4, 6 & 7). Another important finding for our one month L-NNA treatment study is that hemizygotic over-expression of A53T in mice was enough to significantly increase the S-nitrosylation of CK2α and GRK6 (Fig 4). These results suggest that both aging and transgenic expression of A53T α-syn could contribute to PD through increasing nitrosative stress in the brain (Figs 3 & 4).

To determine if the age-dependent increased in S-nitrosylation of GRK6 and CK2α was dependent on nNOS and hence affected the accumulation of pSer129 α-syn (Fig 3), we generated A53T transgenic mice with or without deletion of one copy of nNOS (A53T -/+ and A53T -/+; nNOS-/+). At 6, 9 and 12 months of age, we sacrificed these mice and then performed *in vivo* biotin-switch assay to analyze the level of GRK6, CK2α and GAPDH S-nitrosylation in these mouse brain samples. At the same time, we also measured the level of α-syn and pSer129 α-syn in these samples. We found that reduction of α-syn and pSer129 α-syn was observed in an age-dependent manner in the A53T transgenic mice with the heterogenic deletion of nNOS (A53T -/+; nNOS-/+). It was particular prominent in the 9 and 12 months old double transgenic mice (Figs 6 & 7). We had also observed a significant age-dependent reduction in the S-nitrosylation of CK2α and GAPDH in the 12 month old A53T transgenic mice with the heterogenic deletion of nNOS (A53T -/+; nNOS-/+) (Fig 7). These results further support our hypothesis that that both aging and NO could modulate the levels of pSer129 α-syn and total α-syn in the mouse brain. Another factor that might affect our results was that other kinases might also contribute to the α-syn phosphorylation such as PLKs. In this study, we just focused on CK2α and GRK6 because studies have shown that they are the primarily kinases that phosphorylate α-syn at S129 [21, 27–29] Taken together, the mouse PD model suggested that even heterogenic deletion of nNOS could have a significant effect on the metabolism of α-syn and pSer129 α-syn level in an age-dependent manner. These results also further consolidate the importance of the interaction between nitrosative stress and aging in the pathogenesis of PD. In comparison to the L-NNA treatment, the double mutant (A53T -/+; nNOS-/+) experiment supports the notion that the contribution of the nitrosative stress in the PD is not just depending on nNOS, but also possibly involving the activity of eNOS and iNOS as we know that different NOS activity can affect the pathogenesis of PD from previous studies [53, 54]. One of the important and novel mechanisms finding in this study is that both aging and overexpression of A53T α-syn could increase nitrosative stress in an animal model of PD. Finally, this study suggests that the modulation of nitrosative stress might be a potential therapeutic approach for PD treatment.

## Supporting information

S1 Fig GRK6 enhances the phosphorylation of α-syn phosphorylation at S129 in a dose-dependent manner. pRK5-α-syn and different amounts of pCMV-Tag2B-GRK6 were transfected to HEK293T cells. A dose-dependent enhanced phosphorylation of α-syn phosphorylation at S129 was observed by GRK6.
(EPS)

S2 Fig CK2α enhances the phosphorylation of α-syn phosphorylation at S129 in a dose-dependent manner. pRK5-α-syn and different amounts of pRK5-myc-CK2α were transfected to HEK293T cells. A dose-dependent enhanced phosphorylation of α-syn phosphorylation at S129 was observed by CK2α.
(EPS)

S1 Raw Images (PDF)
