# Correction: S-Nitrosylation of G protein-coupled receptor kinase 6 and Casein kinase 2 alpha modulates their kinase activity toward alpha-synuclein phosphorylation in an animal model of Parkinson’s disease

**DOI:** 10.1371/journal.pone.0235296

**Published:** 2020-06-18

**Authors:** Weiwei Wu, Chun Chau Sung, Peichun Yu, Jiahua Li, Kenny K. K. Chung

In Figs 3–7, there are incorrect symbols in the labels. Please view the correct figures here.

**Fig 3:**
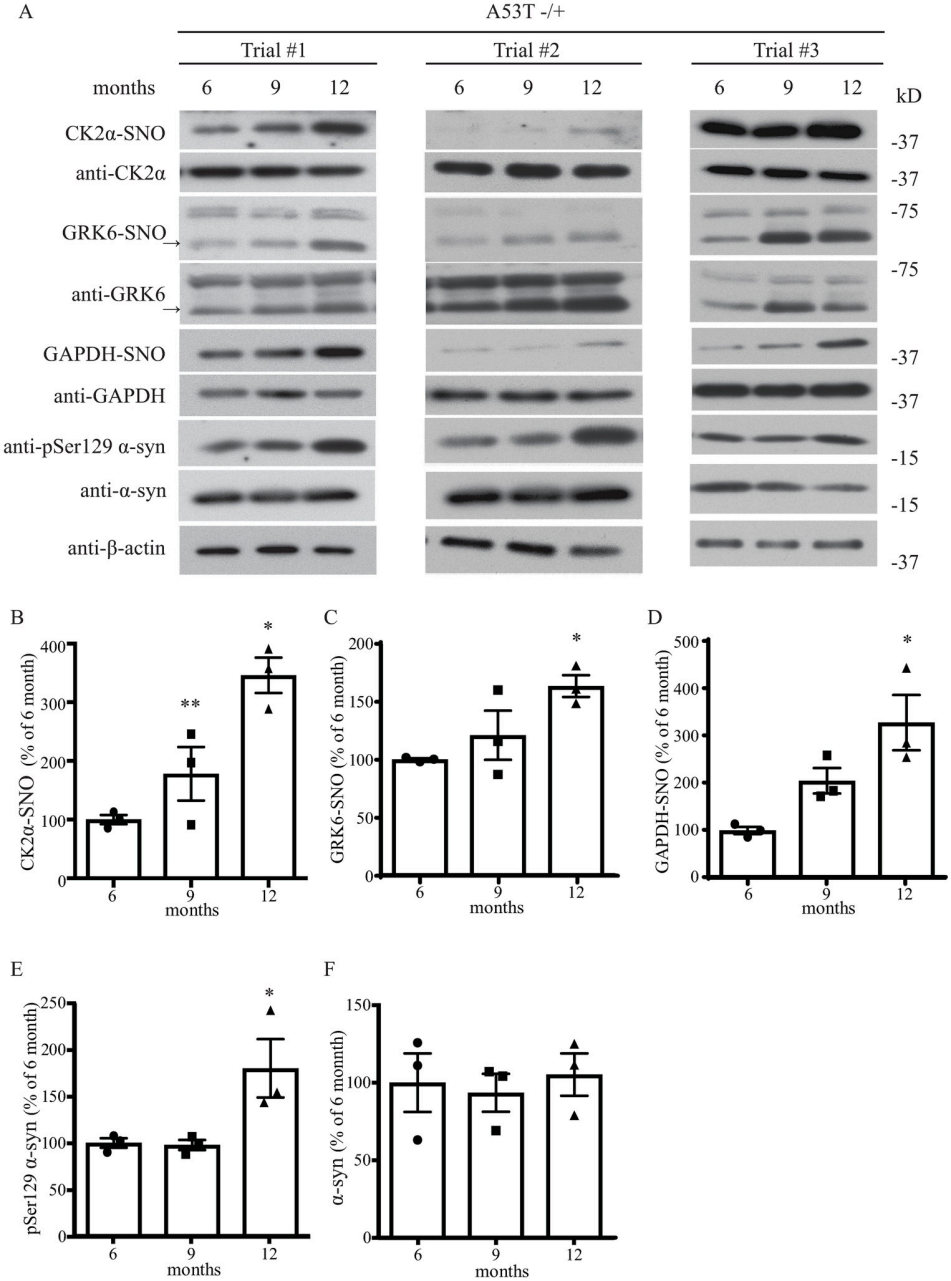
Aging increases GRK6 and CK2α S-nitrosylation in an A53T α-syn transgenic mouse model of PD. (A) Hemizygous A53T α-syn transgenic mouse brain samples of 6, 9 and 12 months old were analyzed with in vivo biotin-switch assay for GRK6, CK2α and GAPDH. The samples were also subject to Western blot analysis of GRK6, CK2α, GAPDH, pSer129 α-syn and α-syn. (→: GRK6 band) (B) Quantification of hemizygous A53T α-syn transgenic mouse brain samples for in vivo CK2α S-nitrosylation as in (A) (* p < 0.05, ** p < 0.01; # of animals = 3 for each time point; one-way ANOVA with Bonferroni post-hoc test). (C) Quantification of hemizygous A53T α-syn transgenic mouse brain samples for in vivo GRK6 S-nitrosylation as in (A) (* p < 0.05; # of animals = 3 for each time point; one-way ANOVA with Bonferroni post-hoc test). (D) Quantification of hemizygous A53T α-syn transgenic mouse brain samples for in vivo GAPDH S-nitrosylation as in (A) (* p < 0.05; # of animals = 3 for each time point; one-way ANOVA with Bonferroni post-hoc test). (E) Quantification of hemizygous A53T α-syn transgenic mouse brain samples for protein levels of pSer129 α-syn as in (A) (# of animals = 3 for each time point). (F) Quantification of hemizygous A53T α-syn transgenic mouse brain samples for protein levels of α-syn as in (A) (# of animals = 3 for each time point).

**Fig 4:**
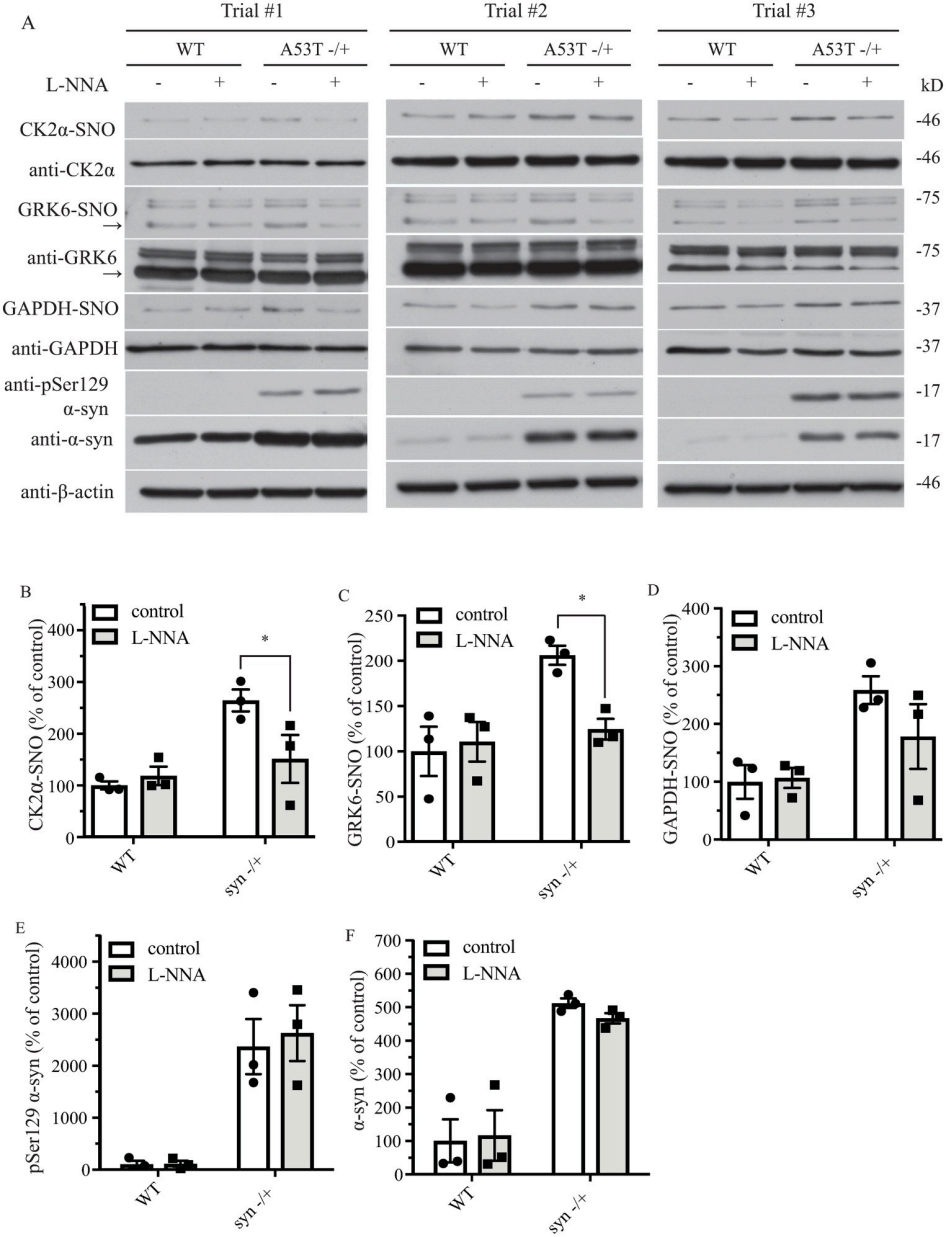
A53T α-syn transgenic expression increases S-nitrosylation of GRK6, CK2α and GAPDH in the mouse brain. (A) WT, and hemizygous A53T α-syn transgenic mouse brain samples of 9 months old treated with or without L-NNA were analyzed with in vivo biotin-switch assay for CK2α and GAPDH. The samples were also subject to Western blot analysis of CK2α, GAPDH, pSer129 α-syn and α-syn. (→: GRK6 band) (B) Quantification of WT and hemizygous A53T α-syn transgenic mouse brain samples for in vivo CK2α S-nitrosylation as in (A) (* p < 0.05; no. of animals = 3 in each group; two-way ANOVA with Bonferroni post-hoc test). (C) Quantification of WT and hemizygous A53T α-syn transgenic mouse brain samples for in vivo GRK6 S-nitrosylation as in (A) (* p < 0.05; # of animals = 3 in each group; two-way ANOVA with Bonferroni post-hoc test). (D) Quantification of WT and hemizygous A53T α-syn transgenic mouse brain samples for in vivo GAPDH S-nitrosylation as in (A). (E) Quantification of WT and hemizygous A53T α-syn transgenic mouse brain samples for protein levels of pSer129 α-syn as in (A) (# of animals = 3 in each group). (F) Quantification of WT and hemizygous A53T α-syn transgenic mouse brain samples for protein levels of α-syn as in (A) (# of animals = 3 in each group).

**Fig 5:**
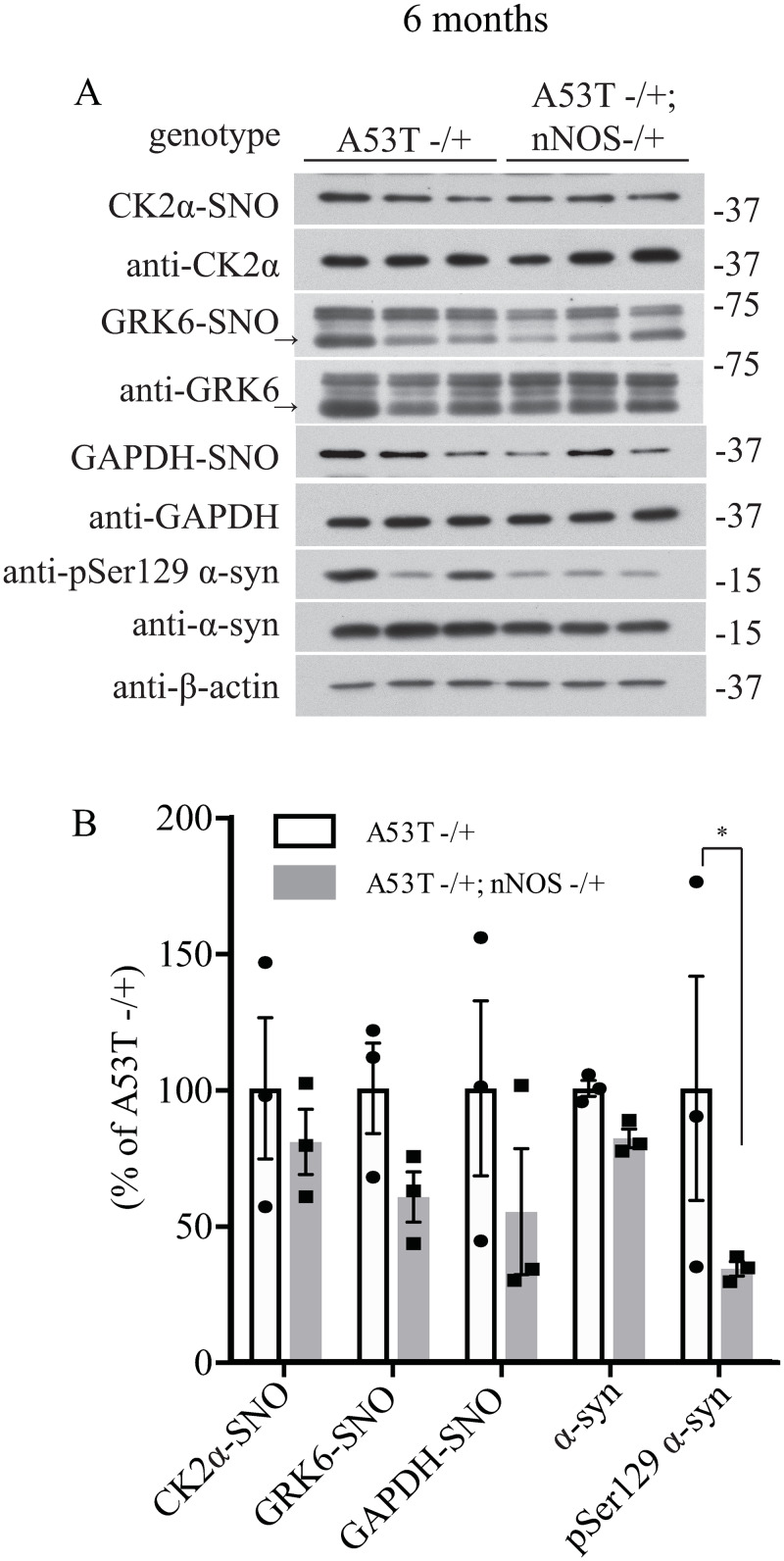
Deletion of neuronal NOS (nNOS) reduces the accumulation of pSer129 α-syn in 6-month-old A53T α-syn transgenic mice. (A) Hemizygous A53T α-syn transgenic mouse brain (A53T -/+), and hemizygous A53T α-syn transgenic and nNOS heterozygous knockout (A53T -/+; nNOS -/+) double mutant mouse brain samples at 6-month-old were analyzed with in vivo biotin-switch assay for CK2α and GAPDH. The samples were also subject to Western blot analysis of CK2α, GAPDH, pSer129 α-syn and α-syn. (→: GRK6 band) (B) Quantification of CK2α, GRK6 and GAPDH S-nitrosylation and protein levels of pSer129 α-syn and α-syn as in (A) (* p<0.05; # of animals = 3 in each group; Student’s t-test).

**Fig 6:**
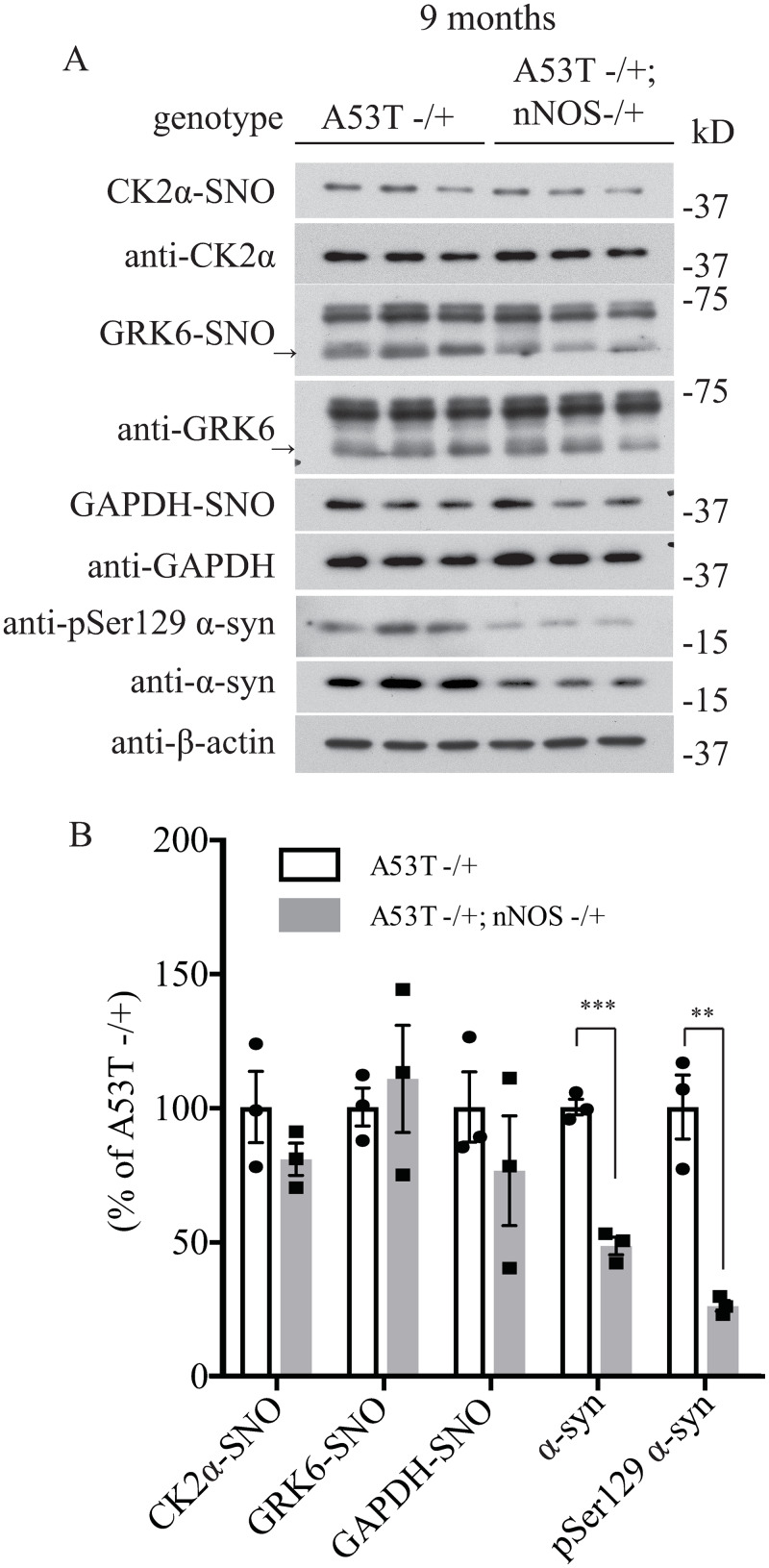
Deletion of neuronal NOS (nNOS) reduces the accumulation of pSer129 α-syn and total α-syn in 9-month-old A53T α-syn transgenic mice. (A) Hemizygous A53T α-syn transgenic mouse brain (A53T -/+), and hemizygous A53T α-syn transgenic and nNOS heterozygous knockout (A53T -/+; nNOS -/+) double mutant mouse brain samples at 9-month-old were analyzed with in vivo biotin-switch assay for CK2α and GAPDH. The samples were also subject to Western blot analysis of CK2α, GAPDH, pSer129 α-syn and α-syn. (→: GRK6 band) (B) Quantification of CK2α, GRK6 and GAPDH S-nitrosylation and protein levels of pSer129 α-syn and α-syn as in (A) (*** p<0.001; ** P<0.01; # of animals = 3 in each group; Student’s t-test).

**Fig 7:**
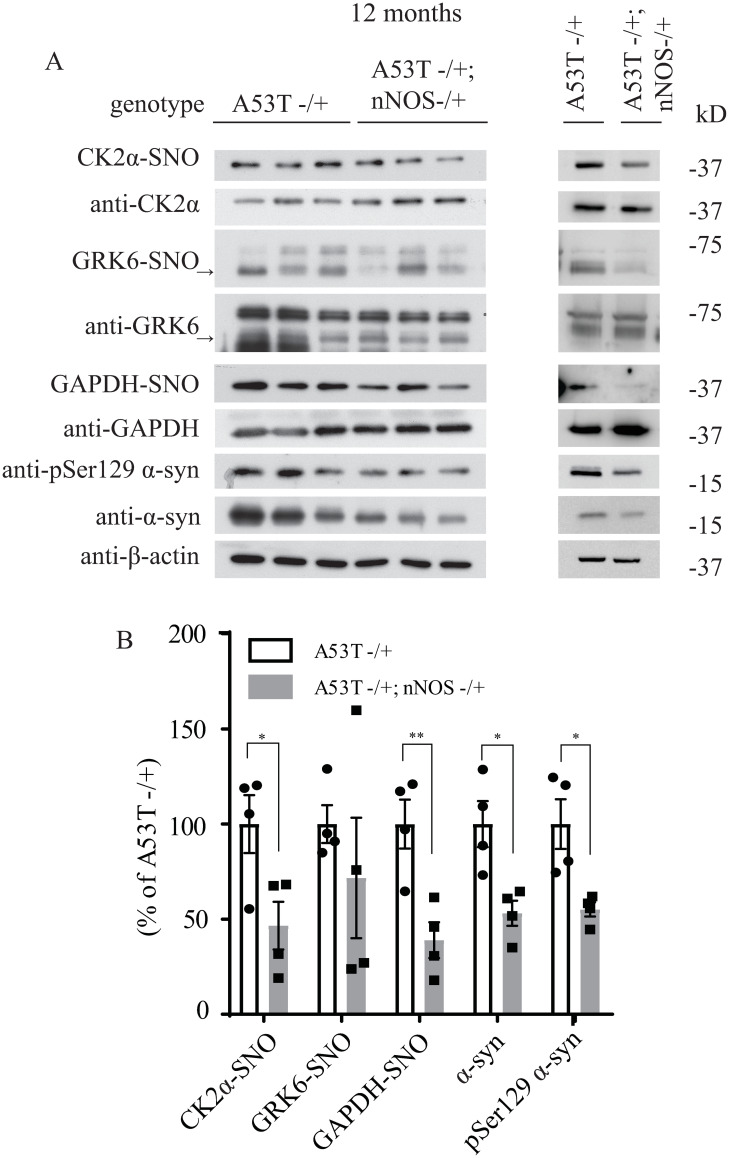
Deletion of neuronal NOS (nNOS) reduces the accumulation of pSer129 α-syn and total α-syn in 12-month-old A53T α-syn transgenic mice. (A) Hemizygous A53T α-syn transgenic mouse brain (A53T -/+), and hemizygous A53T α-syn transgenic and nNOS heterozygous knockout (A53T -/+; nNOS -/+) double mutant mouse brain samples at 12-month-old were analyzed with in vivo biotin-switch assay for CK2α and GAPDH. The samples were also subject to Western blot analysis of CK2α, GAPDH, pSer129 α-syn and α-syn. (→: GRK6 band) (B) Quantification of CK2α, GRK6 and GAPDH S-nitrosylation and protein levels of pSer129 α-syn and α-syn as in (A) (* p<0.05; ** p<0.01; # of animals = 4 in each group; Student’s t-test).
