# Supplementary figures and images for: S-Nitrosylation of G protein-coupled receptor kinase 6 and Casein kinase 2 alpha modulates their kinase activity toward alpha-synuclein phosphorylation in an animal model of Parkinson’s disease
Source: PLoS One. 2020 Apr 28;15(4):e0232019. doi: 10.1371/journal.pone.0232019 (PMC7188290; doi:10.1371/journal.pone.0232019)

Fig 1

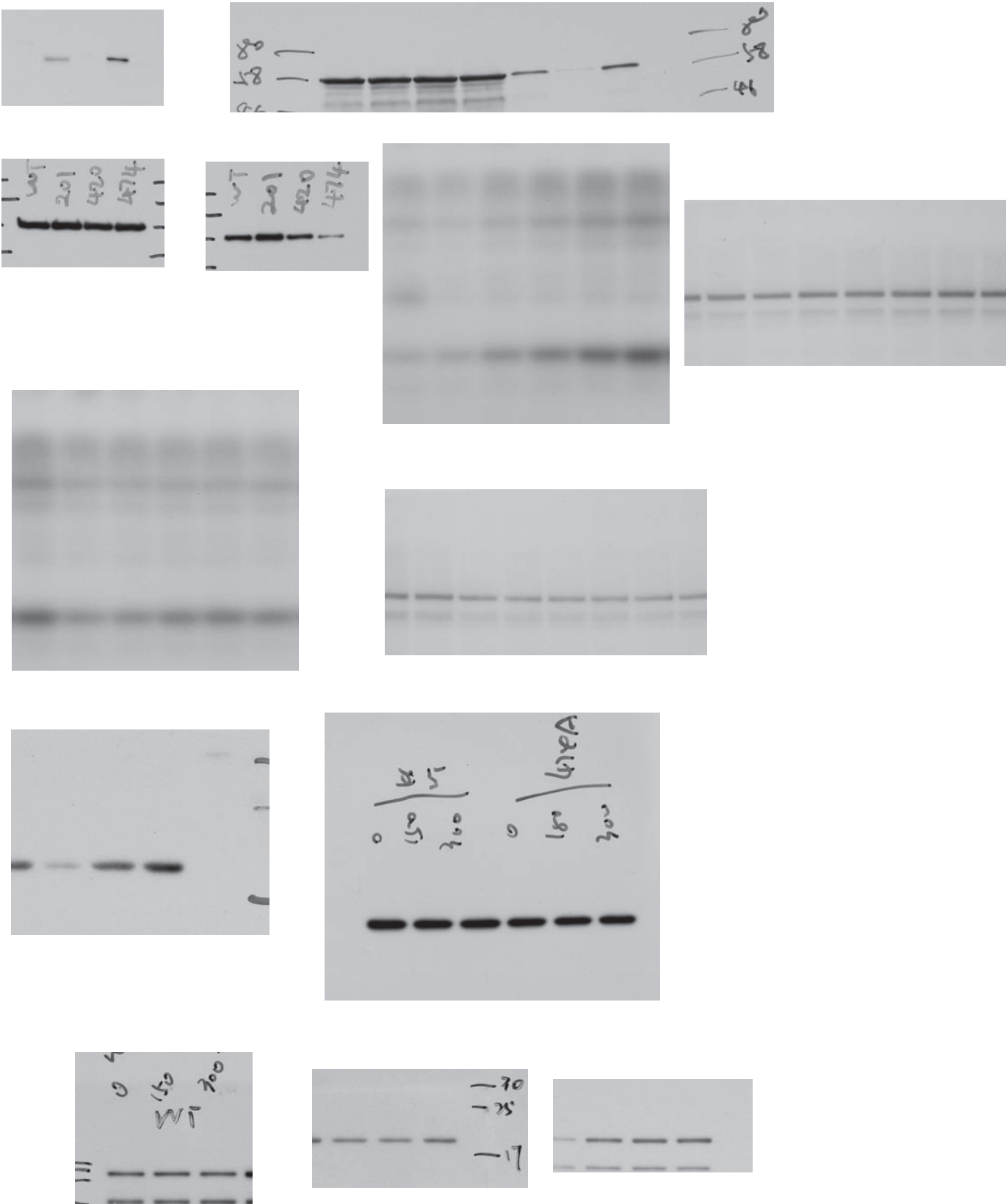

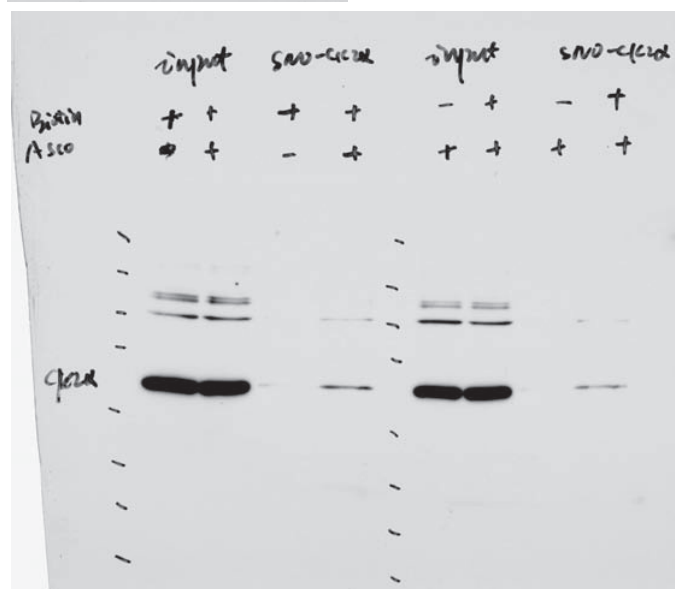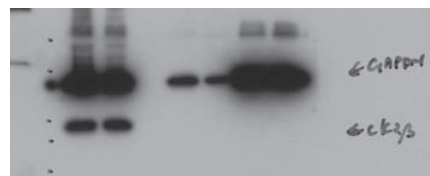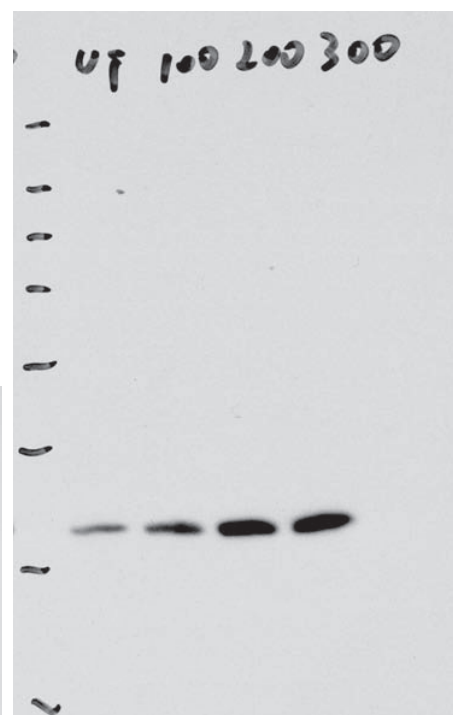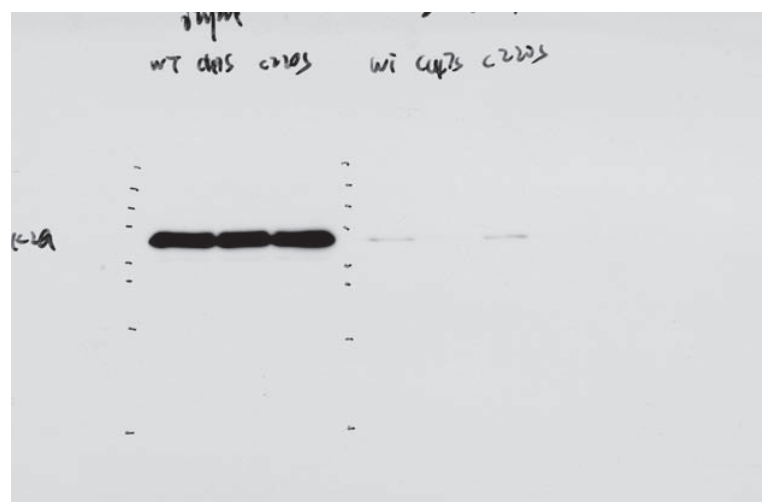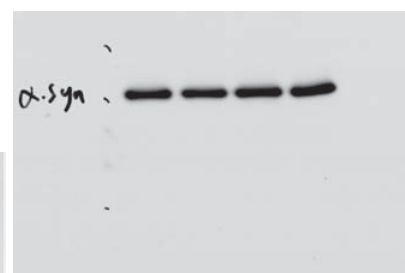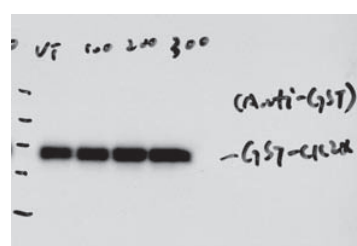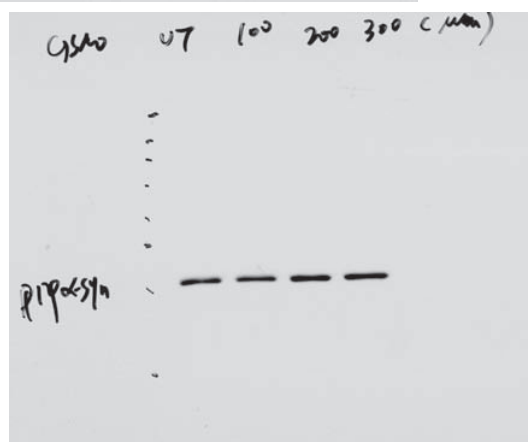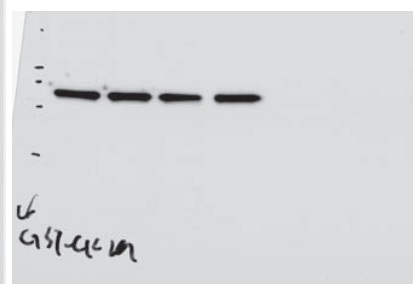

Fig 3A

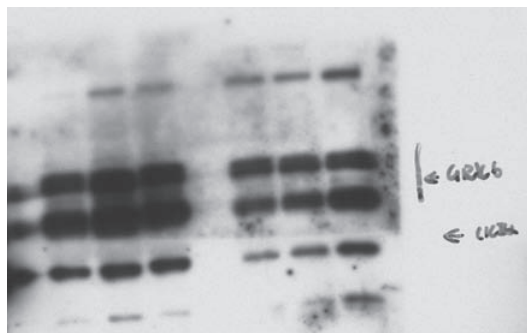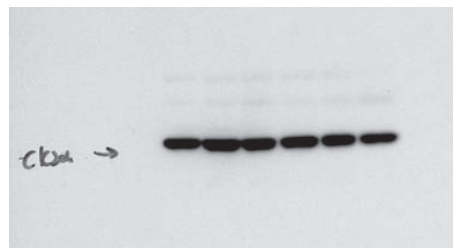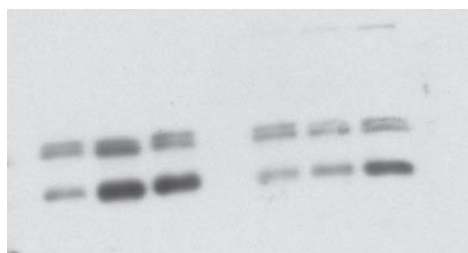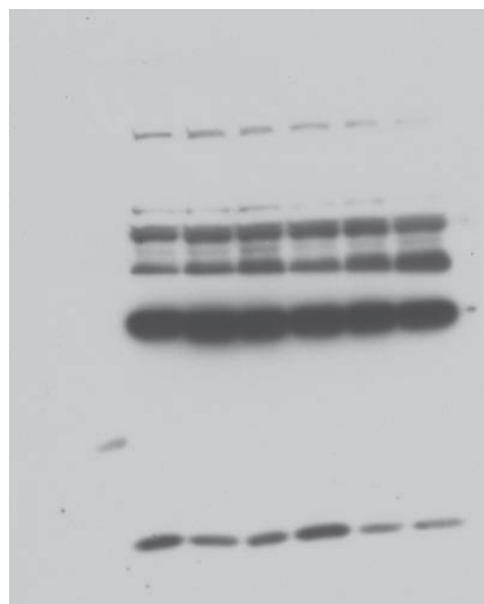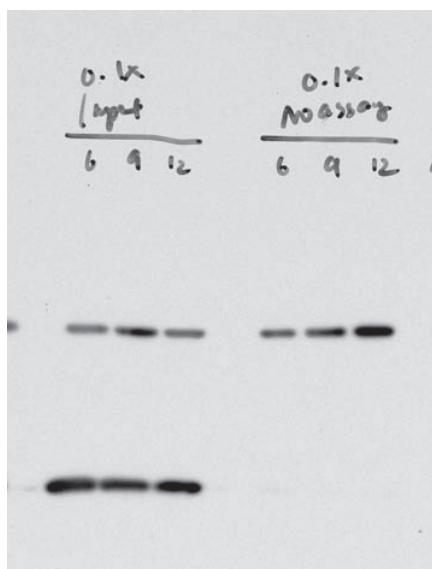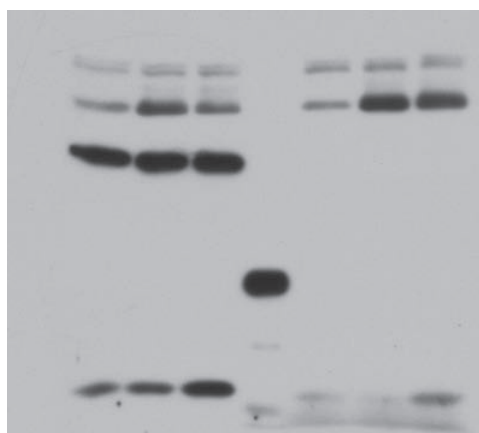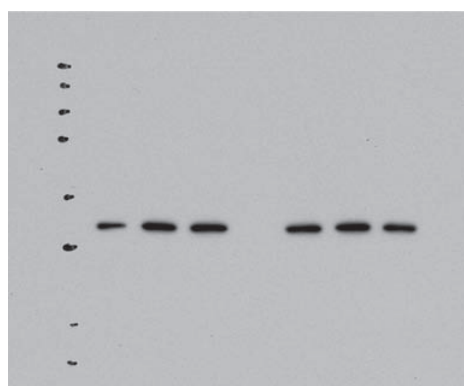

Fig 4A

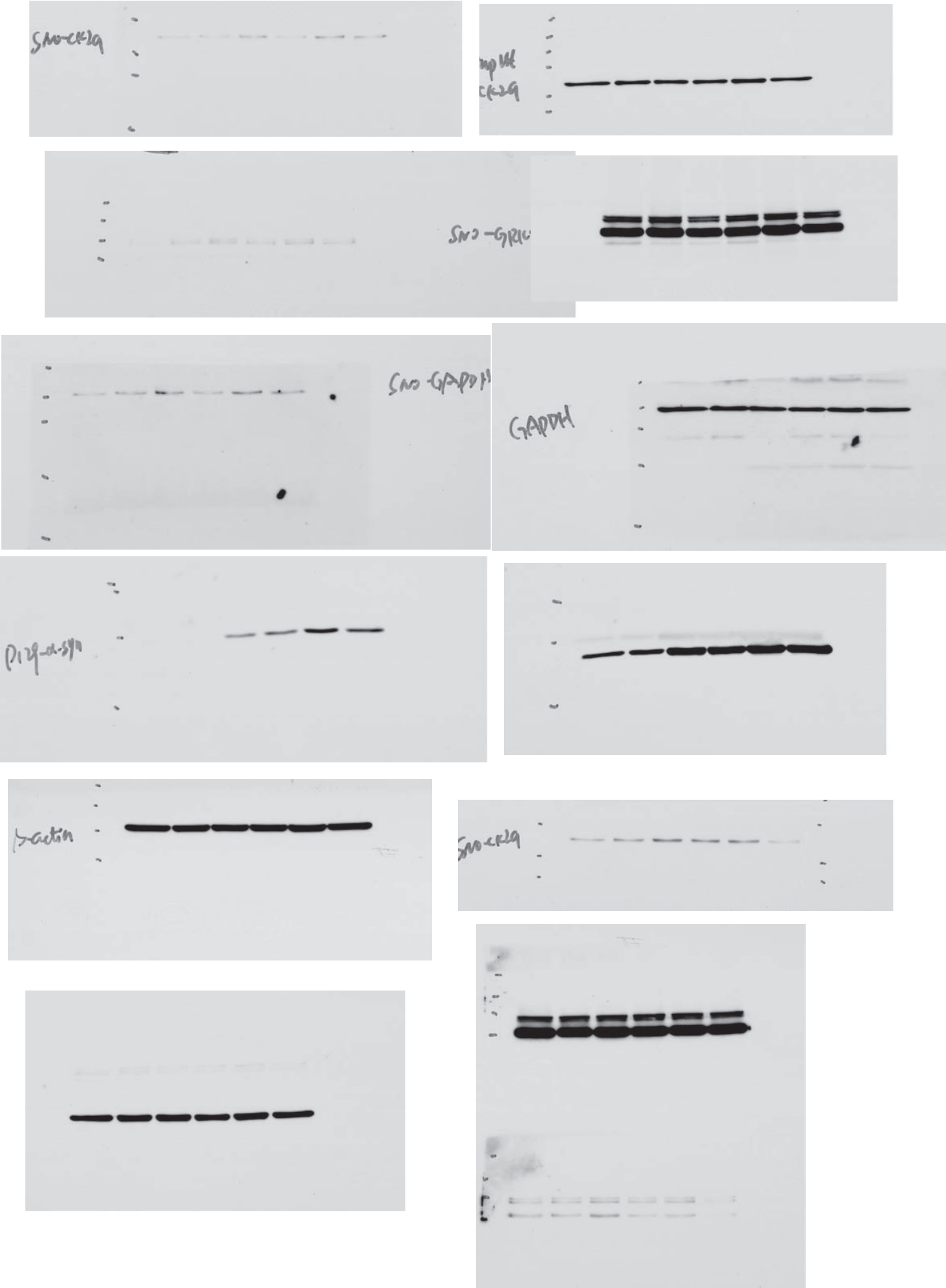

Fig4\_2

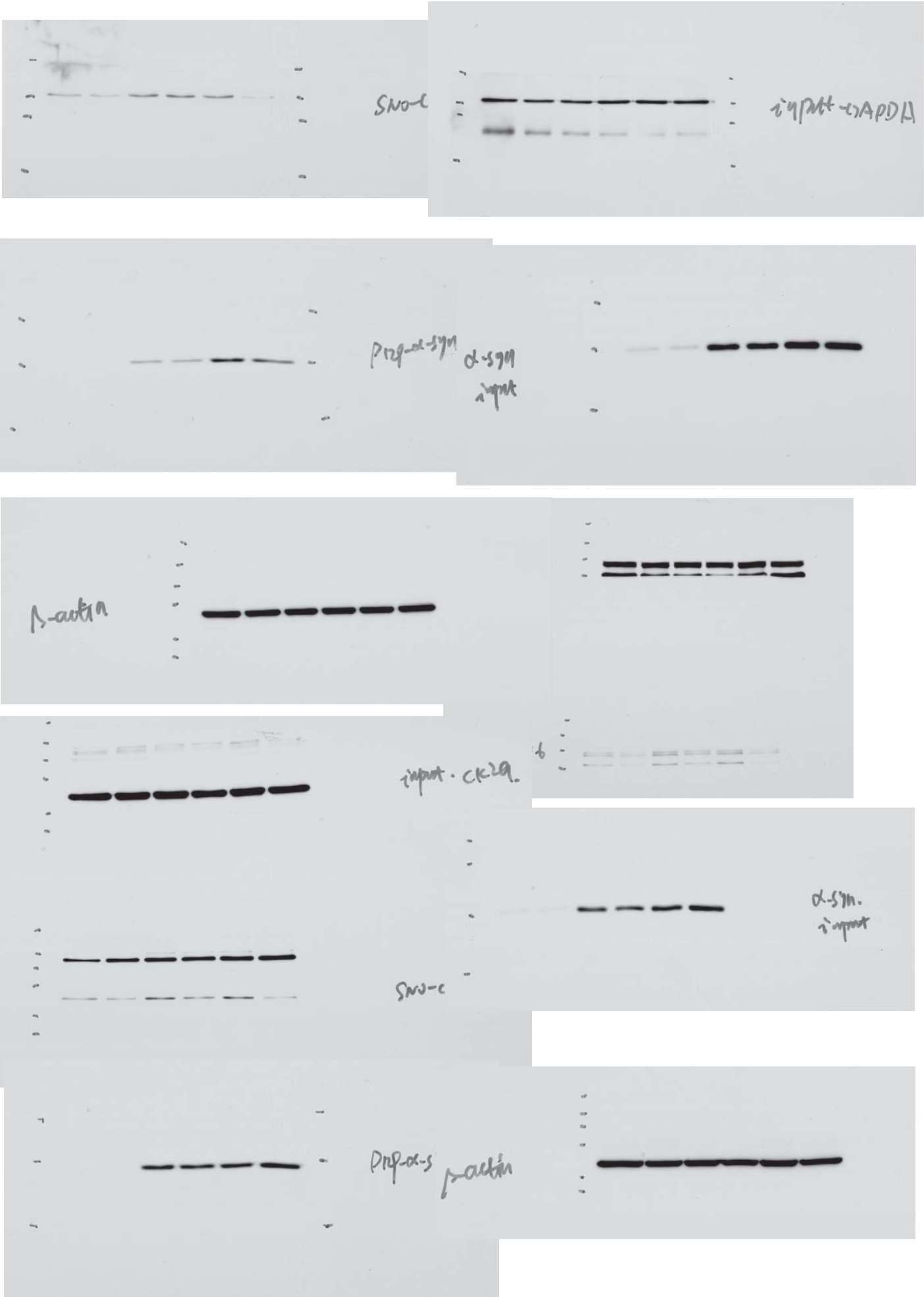

Fig 5A

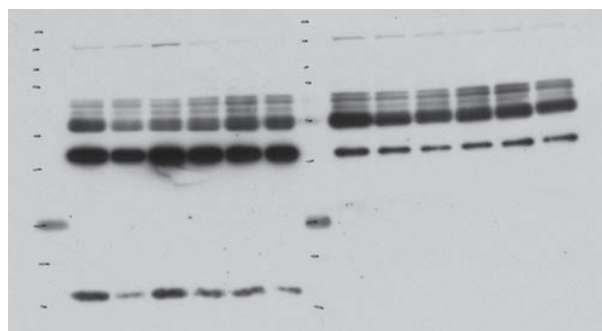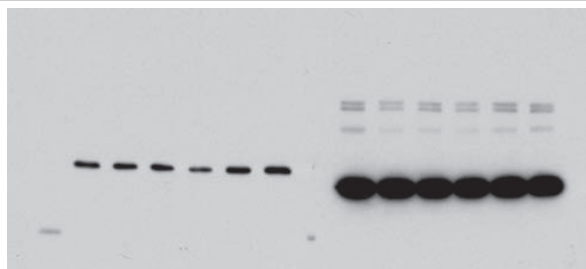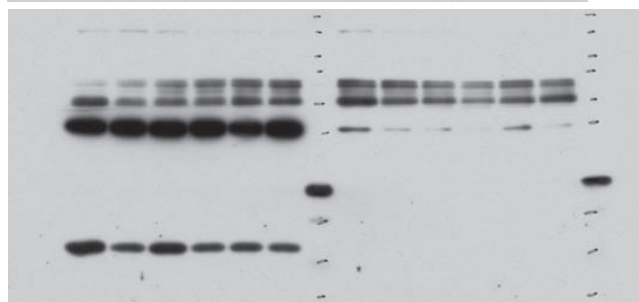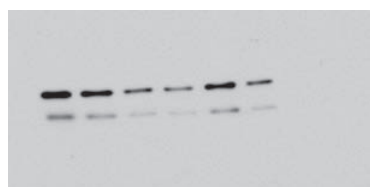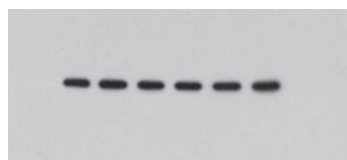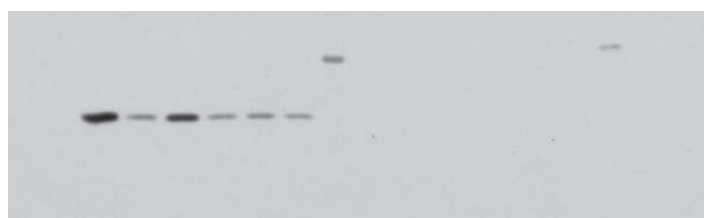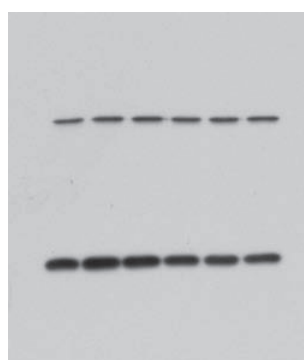

Fig 6A

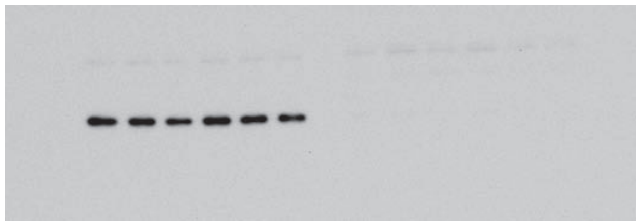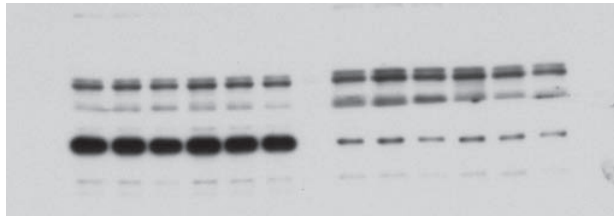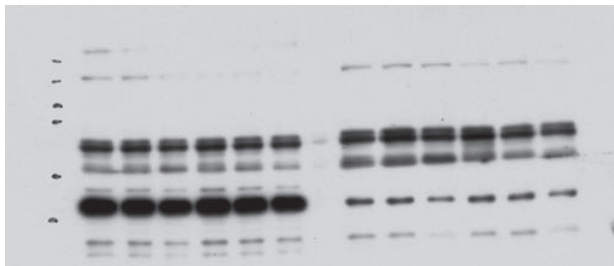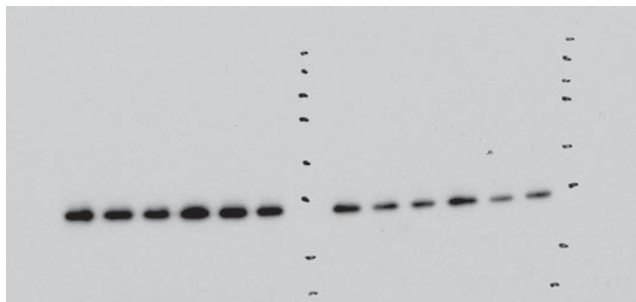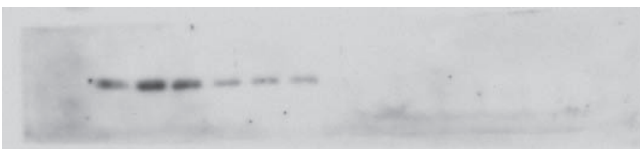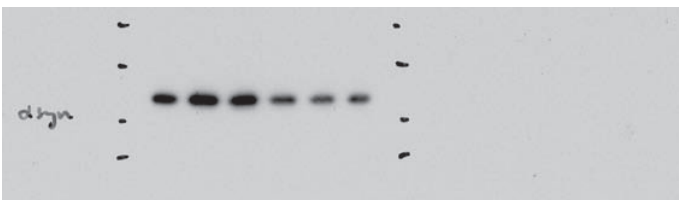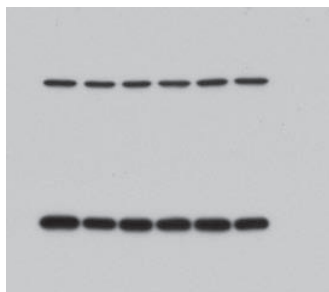

Fig 7A

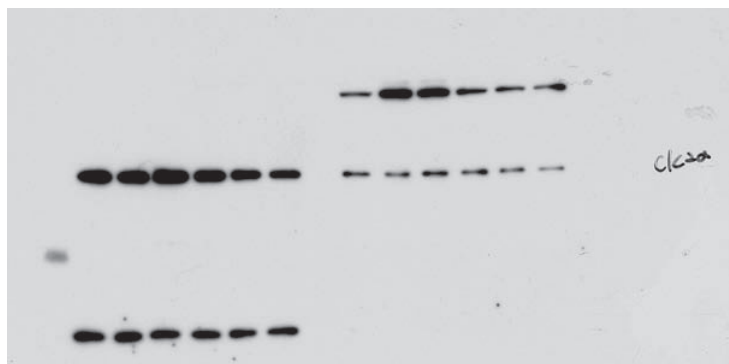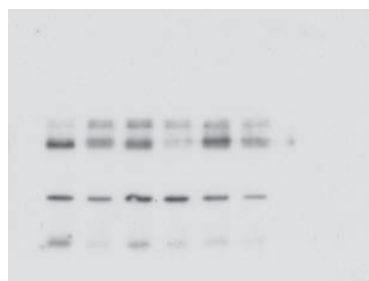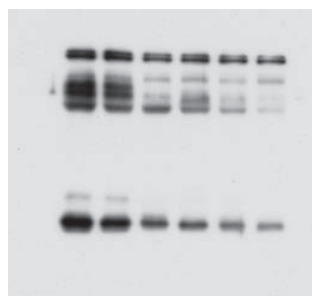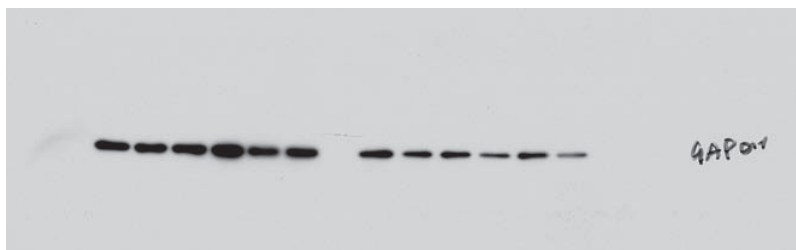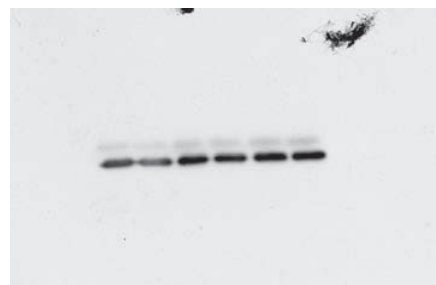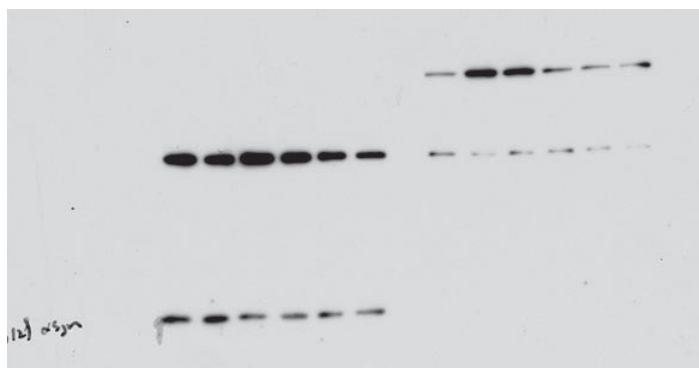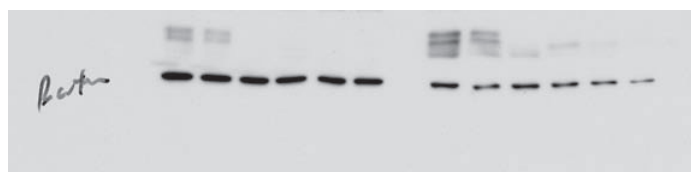

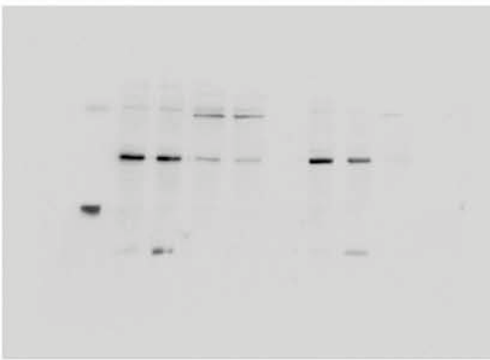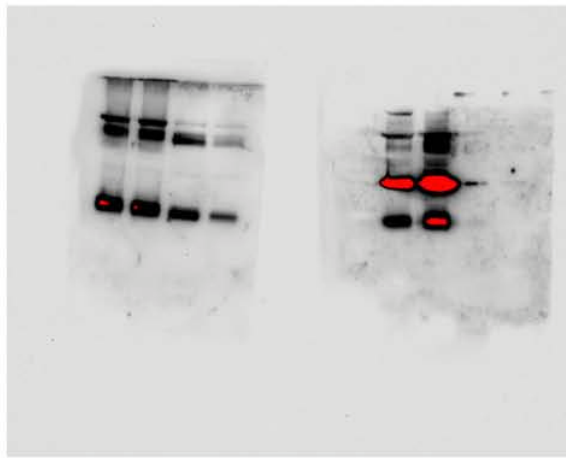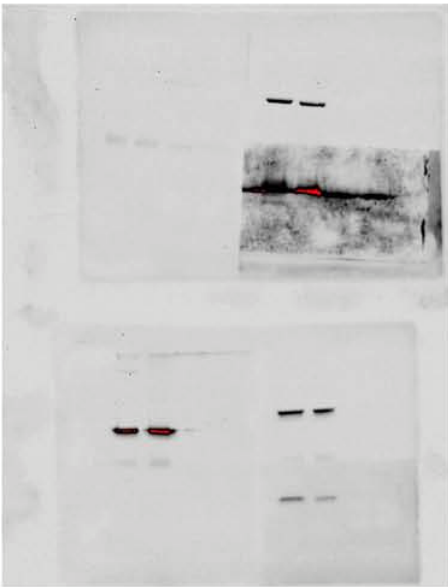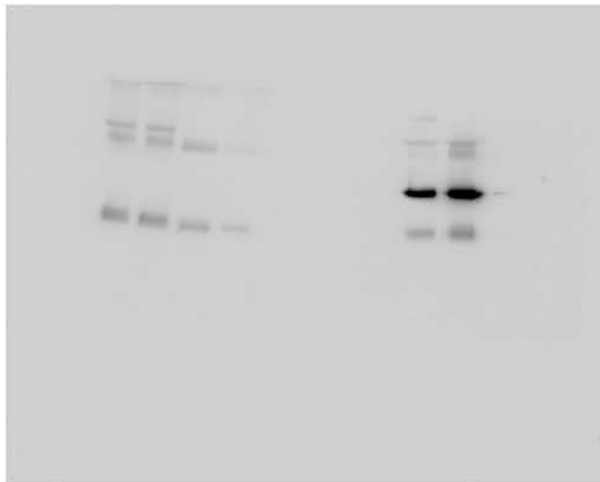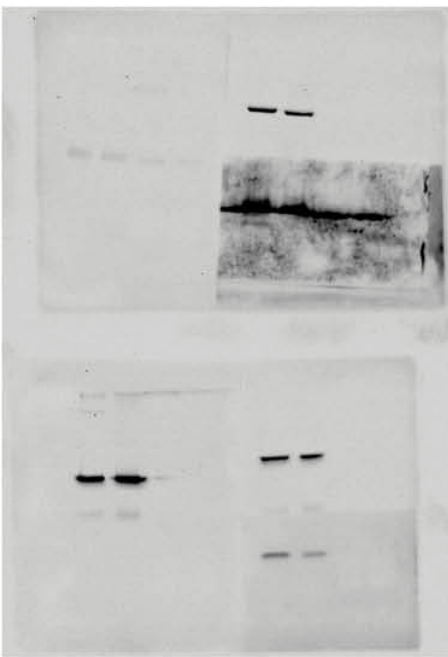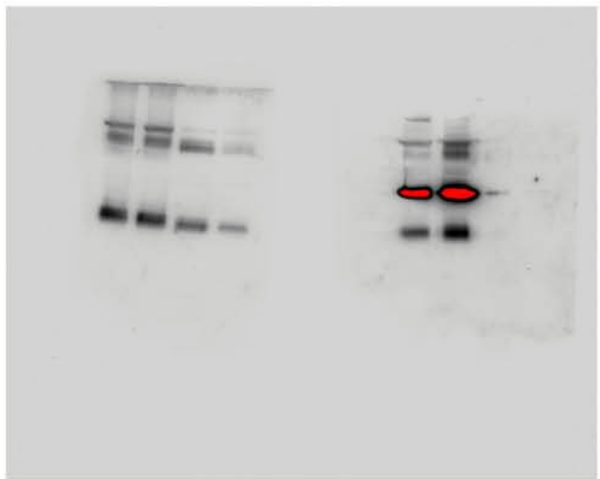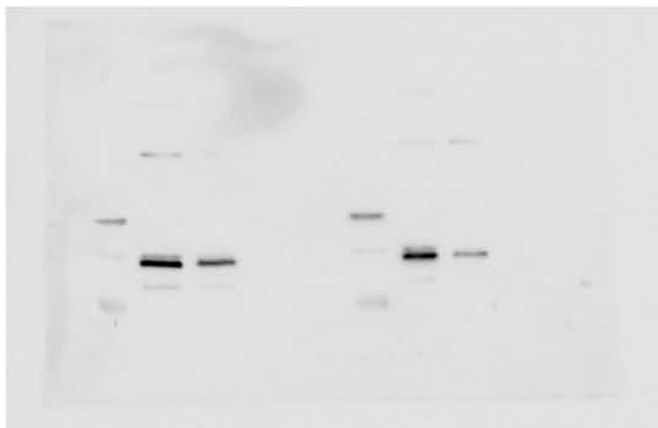

Supp. Fig. 1

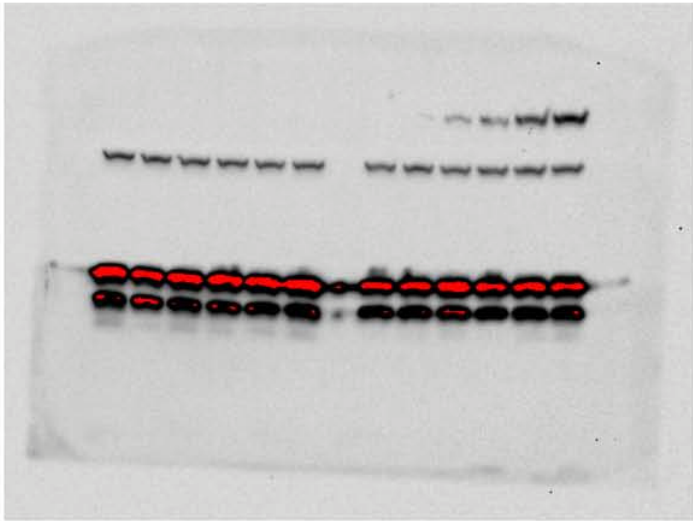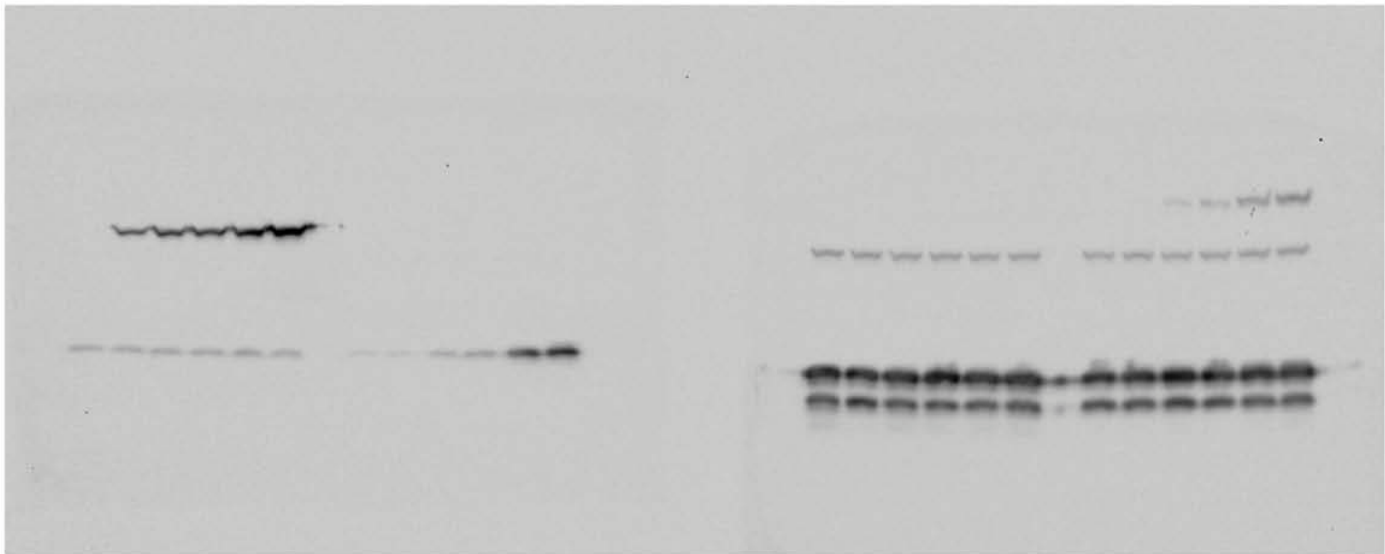

Supplement: S1 Raw Images — (PDF) [file pone.0232019.s003.pdf]
